# Maladaptive activation of Nav1.9 channels by nitric oxide causes triptan-induced medication overuse headache

**DOI:** 10.1038/s41467-019-12197-3

**Published:** 2019-09-18

**Authors:** Caroline Bonnet, Jizhe Hao, Nancy Osorio, Anne Donnet, Virginie Penalba, Jérôme Ruel, Patrick Delmas

**Affiliations:** 1 Aix-Marseille-Université, CNRS, Laboratoire de Neurosciences Cognitives, UMR 7291, CS8011, Bd Pierre Dramard, 13344 Marseille, France; 2 Centre d’évaluation et de traitement de la douleur, Hôpital de la Timone, Marseille, France

**Keywords:** Diseases, Neurological disorders, Headache, Migraine

## Abstract

Medication-overuse headaches (MOH) occur with both over-the-counter and pain-relief medicines, including paracetamol, opioids and combination analgesics. The mechanisms that lead to MOH are still uncertain. Here, we show that abnormal activation of Nav1.9 channels by Nitric Oxide (NO) is responsible for MOH induced by triptan migraine medicine. Deletion of the *Scn11a* gene in MOH mice abrogates NO-mediated symptoms, including cephalic and extracephalic allodynia, photophobia and phonophobia. NO strongly activates Nav1.9 in dural afferent neurons from MOH but not normal mice. Abnormal activation of Nav1.9 triggers CGRP secretion, causing artery dilatation and degranulation of mast cells. In turn, released mast cell mediators potentiates Nav1.9 in meningeal nociceptors, exacerbating inflammation and pain signal. Analysis of signaling networks indicates that PKA is downregulated in trigeminal neurons from MOH mice, relieving its inhibitory action on NO-Nav1.9 coupling. Thus, anomalous activation of Nav1.9 channels by NO, as a result of chronic medication, promotes MOH.

## Abstract (web summary)

The precise mechanisms that lead to medication-overuse headaches (MOH), which can occur with both over-the-counter and prescription pain-relief medicines, are still uncertain. In this study, authors show that the abnormal activation of dural nociceptor Nav1.9 channels by Nitric Oxide is responsible for triptan-induced MOH, causing hyperexcitability of dural nociceptors and headache.

## Introduction

Chronic headache encompasses many different headache diagnoses and include chronic migraines, chronic tension-type headaches, medication-overuse headaches (MOH), and other types of daily persistent headaches. Migraine, a frequently incapacitating neurovascular disorder, affects hundreds of millions of individuals worldwide^1^. It is characterized by a severe, debilitating and throbbing unilateral headache accompanied by a host of neurological symptoms including hypersensitivity to visual and auditory stimulation, nausea and vomiting, and a variety of autonomic, cognitive and motor disturbances^2,3^.

Current antimigraine drugs target trigeminovascular 5-HT_1B/1D_, 5-HT_1F_, and CGRP receptors. These different antimigraine medications induce adverse side effects, including MOH, which is a worldwide health problem with a prevalence range of 1–2% with a 3:1 female to male ratio. MOH is a severe form of headache where the patients are prone to develop primary headaches with unsuccessful treatments. Patients with migraine or tension-type headaches have a higher potential for developing MOH than other primary headaches. MOH does not develop in persons without a history of headache when medication is being used for other conditions, such as inflammatory diseases. In addition, virtually all medication for headaches may lead to MOH, including opioids, ergotamine, butalbital-containing medicine, triptans or a combination thereof. Thus, MOH is generated in headache-prone persons by the interaction between headache medication and pre-existing headache disorder pathways.

The precise mechanisms that lead to MOH development are largely unknown. However, multiple factors may be implicated, including genetic predisposition, sensitization within the trigeminal (TG) system, abnormal cortical pain processing and decreased antinociceptive activity of the supraspinal structures^4–7^.

Multiple studies indicate that migraine medication induces sensitization of peripheral and central pain pathways. For instance, chronic use of opioids and triptans in animals has been shown to increase the level of calcitonin gene-related peptide (CGRP), which is involved in neurogenic inflammation and headache pain^8,9^. These animals develop a persistent hypersensitivity or latent sensitization to provocative triggers, such as environmental stress stimulus and the well-known human migraine trigger nitric oxide (NO). This latent sensitization persists long after discontinuation of drug administration and produce a state of generalized cutaneous allodynia that was detected in periorbital regions and hind paw.

To probe molecular mechanisms that lead to MOH, we developed a MOH mouse model based on the sustained administration of sumatriptan, a 5-HT receptor agonist selective for 5-HT_1D_ and 5-HT_1B_ subtypes. We applied the MOH model to transgenic mice for studying the role of the nociceptor-specific voltage-gated Nav1.9 channel in headache. The function of this channel in the TG pain pathway is still poorly understood^10–12^. Nav1.9 channel is known to generate a persistent, tetrodotoxin (TTX)-resistant Na^+^ current that promotes sustained neuronal activity in dorsal root ganglion (DRG) neurons^13–17^. Nav1.9 is known to contribute to both inflammatory^18–22^ and neuropathic^22,23^ somatic pain in animal models, and variants of the *Scn11a* gene encoding Nav1.9 in humans lead to congenital insensitivity to pain and to painful syndromes^24–27^.

Using molecular, electrophysiological, and behavioral approaches, we show that mice chronically treated with sumatriptan display increased responsiveness of Nav1.9 to NO, leading to headache/migraine-like symptoms including generalized allodynia, photophobia, and phonophobia. MOH mice show deficit in PKA-mediated inhibition of NO–Nav1.9 coupling, causing hyperactivity of meningeal nociceptors and inflammation in the meninges. Thus, our data identify abnormal activation of Nav1.9 by NO as a central determinant of triptan-induced MOH and pave the way for the development of mechanism-based treatment strategies that can improve the management of primary headaches.

## Results

### Nav1.9 is expressed in meningeal nociceptors

**Fig. 1:**
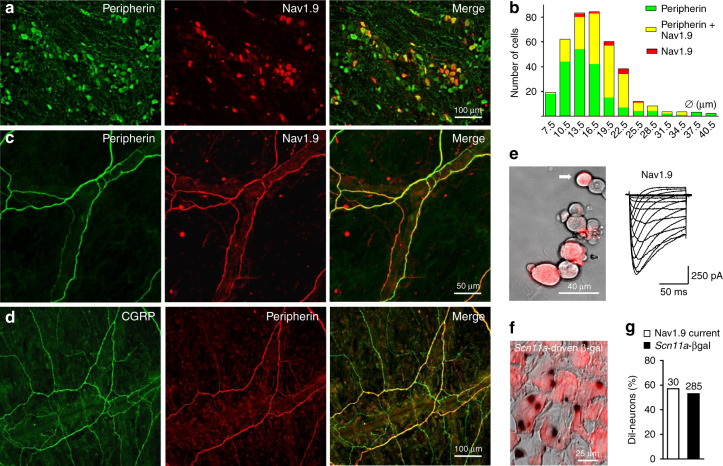
Nav1.9 is expressed in meningeal nerve fibers and dural afferent neurons. **a** Cryosections of a mouse TG were double-labeled for peripherin and Nav1.9. Images are projections of seven consecutive optical sections spanning 9 µm. Rightmost panel: merged image. **b** Histogram showing the distribution of TG neurons immunopositive for peripherin (green), Nav1.9 (red), or both (yellow) as a function of cell body diameter (⌀). Bin size = 3 µm. **c** Mouse whole-mount of dura mater was double-labeled for peripherin and Nav1.9. Images are projections of 16 consecutive optical sections spanning 22 µm. Rightmost panel: merged image. **d** Immunostaining for CGRP and peripherin in mouse dura mater. Images are projections of 21 consecutive optical sections spanning 23 µm. Rightmost panel: merged image. **e** Left panel: TG neurons cultured 2 days after DiI application through a cranial window in the parietal bone. This is a composite image of two different fields of the same culture dish indicated by a white box. Right panel: recording of Nav1.9 current in the retrogradely labeled TG neuron indicated by an arrow. The current was evoked by 100 ms-voltage steps from −80 to −5 mV from a Vh of −100 mV. CsF-containing patch pipette solution. **f** Image from a *Scn11a*^*lacZ*^ mouse showing β-galactosidase expression (dark dots) in DiI^+^ afferent neurons (red) from the TG. **g** Histogram showing the percentage of DiI^+^ afferent neurons exhibiting Nav1.9 current (patch clamp) or β-galactosidase (staining)

Immunostaining of mouse TG cryosections indicated that Nav1.9 is expressed in 32% of TG neurons (Fig. 1a). Nearly all immunopositive neurons (96%, *n* = 174) exhibited small-diameter or medium-diameter cell bodies (<27 µm, average largest diameter ⌀ = 17.4 ± 0.3 µm) (Fig. 1b), while a minority of them (3.8%, *n* = 7 out of 181) displayed large cell bodies (⌀ ≥ 27 µm). Small peripherin-positive fibers showed features of apposition with meningeal arteries in whole mount of mouse dura mater (Supplementary Fig. 1A). Dual-labeling showed that 89% (*n* = 124/139) and 91% (*n* = 101) of peripherin-positive meningeal fibers were immunoreactive for Nav1.9 and CGRP, respectively (Fig. 1c, d), suggesting that Nav1.9 and CGRP co-distribute in a large proportion of meningeal fibers. Double-labeling for Nav1.9 and CGRP could not be made due to the different fixation conditions of primary antibodies. However, the quasi-totality of β-gal -positive TG neurons exhibited CGRP staining in cryosections from *Scn11a*-GAL reporter transgenic mice (*n* = 63/64) (Supplementary Fig. 1B). Few Nav1.9-positive meningeal fibers were also found to be immunoreactive for NF200, possibly corresponding to some lightly myelinated sensory fibers (Supplementary Fig. 1C, D). A majority (57%) of retrogradely labeled (DiI) dural afferent neurons was found to express Nav1.9 current using patch clamp recording (Fig. 1e, g). Consistently, 54% of DiI^+^-dural afferent neurons from *Scn11a*-GAL reporter transgenic mice exhibited β-gal enzymatic activity (Fig. 1f, g). Together, these data provide evidence that about half of dural afferent neurons expresses functional Nav1.9 channels.

### *Scn11a* gene inactivation abrogates NO-induced allodynia in MOH mice

**Fig. 2:**
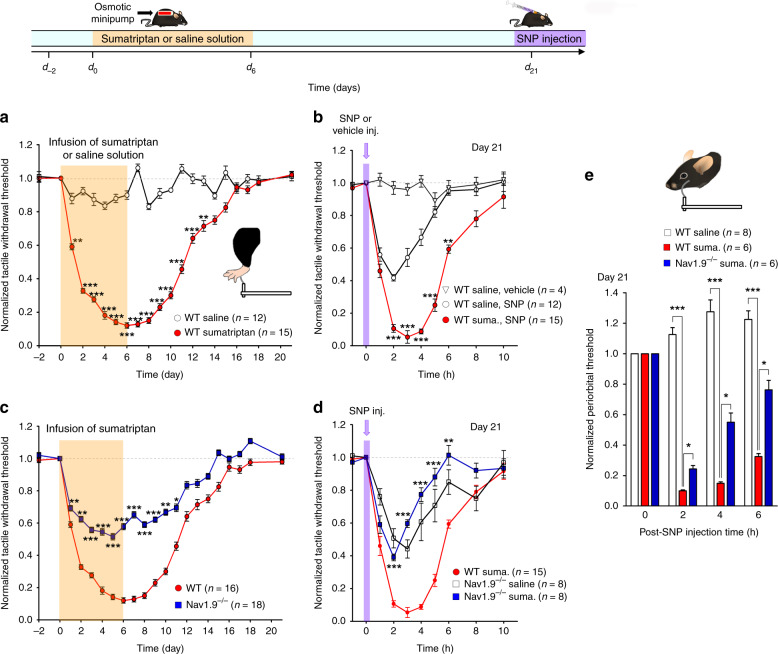
Deletion of Nav1.9 prevents NO-induced generalized allodynia in MOH mice. **a** Infusion of sumatriptan (0.6 mg/kg/day), but not saline solution (0.9%), decreases withdrawal thresholds to tactile stimuli applied to the hind paws of WT mice. Hind paw withdrawal threshold was tested using von Frey filaments (inset). **p < 0.01, ***p < 0.001 compared to saline with Mann–Whitney non-parametric test. Top inset: schematic of mouse treatment over time. **b** Changes in mechanical withdrawal thresholds of the hind paws induced by injection of SNP (0.03 mg/kg) in WT mice pre-treated (red symbols) or not (open circles) with sumatriptan. Data illustrated depict SNP responses 21 days after minipump implantation. **p < 0.01, ***p < 0.001 compared to WT saline, SNP with Mann–Whitney non-parametric test. **c** Hind paw withdrawal responses of sumatriptan-treated Nav1.9^−/−^ mice compared with sumatriptan-treated WT littermates. *p < 0.05, **p < 0.01, ***p < 0.001 compared to WT with Mann–Whitney non-parametric test. **d** Comparison of SNP-induced changes in hind paw withdrawal thresholds in sumatriptan-treated WT mice (red symbols), saline-treated Nav1.9^−/−^ mice (open squares) and sumatriptan-treated Nav1.9^−/−^ mice (blue squares). All tests were made at day 21. **p < 0.01, ***p < 0.001 compared to WT sumatriptan with two-way ANOVA followed by Student–Newman–Keuls test. **e** Normalized periorbital withdrawal threshold plotted as a function of time after SNP injection in saline-treated WT mice (open bars), sumatriptan-treated WT mice (red bars) and sumatriptan-treated Nav1.9^*−/−*^ mice (blue bars). Data illustrated depict SNP responses 21 days after pump implantation. *p < 0.05, ***p < 0.001; two-way ANOVA followed by Student–Newman–Keuls test

To probe the role of Nav1.9 channels in MOH, we developed a model of MOH in Nav1.9^−/−^ mice and their wild-type (WT) littermates using triptan medicines and assessed quantitatively behavioral correlates of headache and migrainous symptoms, including generalized allodynia, photophobia, and phonophobia. Osmotic minipump infusion of sumatriptan in WT mice for 6 days (0.6 mg/kg/day) produced a time-dependent reduction in mechanical withdrawal thresholds of the hind paw relative to saline-treated WT mice (Fig. 2a). Recovery of paw sensory thresholds to sumatriptan pre-infusion values occurred within 18–20 days after minipump implantation (Fig. 2a). Latent sensitization to the migraine trigger NO was tested at day 21, once sensory thresholds were returned to pre-sumatriptan baseline levels (Fig. 2b). Injection of the NO donor sodium nitroprusside (SNP, 0.03 mg/kg) into the loose skin over the neck evoked strong extracephalic tactile allodynia in sumatriptan-treated WT mice compared with saline-treated WT mice (Fig. 2b). Heightened SNP-induced allodynia in sumatriptan-treated WT mice was still observable up to 46 days after minipump implantation (Supplementary Fig. 2A). Heightened mechanical allodynia induced by SNP injection at day 21 was absent in sumatriptan-treated Nav1.9^−/−^ mice (Fig. 2d). Moreover, saline-treated Nav1.9^−/−^ and WT mice displayed similar SNP-induced (basal) allodynia (Fig. 2b, d), indicating that Nav1.9 contributes to the SNP-induced heightened allodynia in sumatriptan-treated WT mice, but plays no apparent role in SNP-induced basal allodynia in saline-treated animals.

Because females have increased risk of developing migraine and MOH, we tested whether infusion of sumatriptan for 6 days (0.6 mg/kg/day) produced mechanical hypersensitivity and latent sensitization to NO in WT female mice as observed for the opposite gender. Chronic sumatriptan produced a strong reduction in mechanical withdrawal thresholds of the hind paw relative to saline-treated WT female mice and to sumatriptan-treated Nav1.9^*−/−*^ female mice (Supplementary Fig. 2B). Injection of SNP (0.03 mg/kg) at day 21 once sensory thresholds had returned to pre-sumatriptan baseline values caused significantly stronger mechanical allodynia in sumatriptan-treated WT female mice compared to saline-treated WT female mice (Supplementary Fig. 2C). SNP-induced heightened mechanical allodynia was absent in sumatriptan-treated Nav1.9^*−/−*^ female mice (Supplementary Fig. 2D), reaching similar amplitude to that caused by SNP in saline-treated Nav1.9^*−/−*^ female mice (Supplementary Fig. 2D). This series of experiments shows that Nav1.9, as observed in male mice, had no role in SNP-induced allodynia in saline-treated animals, but contributes to the heightened SNP allodynia in sumatriptan-treated female mice.

SNP injection at day 21 was also found to reduce mean periorbital von Frey thresholds (periorbital allodynia) in sumatriptan-treated WT male mice compared with saline-treated WT male mice (Fig. 2e). Deletion of Nav1.9 significantly attenuated the SNP-induced periorbital allodynia in sumatriptan-treated animals (Fig. 2e). Altogether, these data indicate that Nav1.9 contributes to NO-induced cephalic and extracephalic tactile allodynia in MOH mice.

### Lack of NO-induced visual and auditory symptoms in Nav1.9 KO mice

**Fig. 3:**
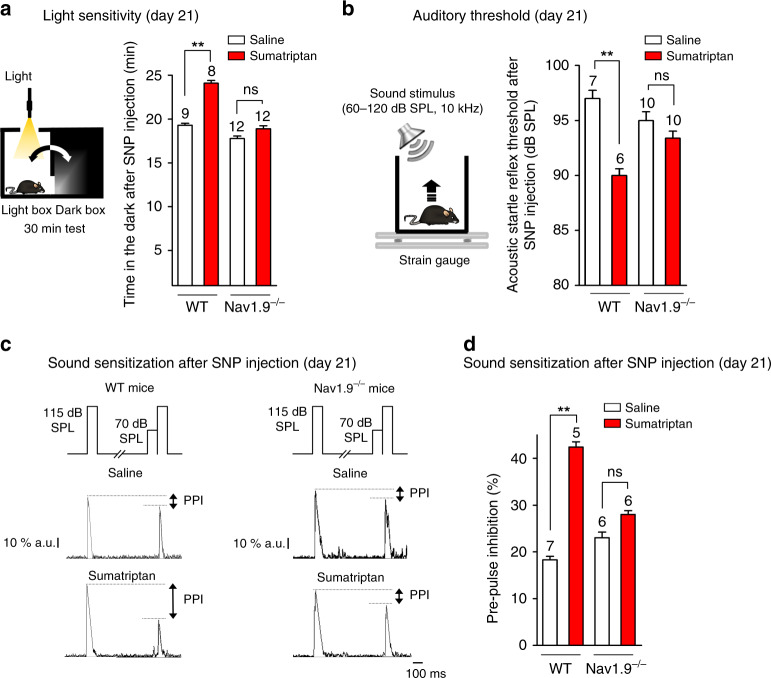
Deletion of Nav1.9 prevents NO-induced photophobia and phonophobia in MOH mice. **a** Light sensitivity of sumatriptan-treated and saline-treated WT and Nav1.9^*−/−*^ mice 2 h after injection of SNP (0.03 mg/kg) at day 21. Visible light: 380–740 nm, 2600 lx. Behavioral tests were made for a duration of 30 min. ns not significant; **p < 0.01; Mann–Whitney test. **b** ASR threshold in sumatriptan-treated and saline-treated WT and Nav1.9^*−/−*^ mice 2 h after injection of SNP at day 21. ns not significant; **p < 0.01; Mann–Whitney test. **c** SNP-induced pre-pulse inhibition: the PPI was measured using the protocol illustrated (top panels) in saline- (middle panel) and sumatriptan- (bottom panel) WT and Nav1.9^*−/−*^ mice 2 h after injection of SNP. a.u. arbitrary unit. **d** Comparison of the PPI in sumatriptan-treated or saline-treated WT and Nav1.9^*−/−*^ mice 2 h after SNP injection. Tests were made at day 21. ns not significant; **p < 0.01; Mann–Whitney test

Besides pain, disabling symptoms of MOH often include photophobia and/or phonophobia^28^. To evaluate light-aversive behavior (photophobia) of mice after SNP injection, we used the light–dark transition test^29^. Two hours after SNP injection, sumatriptan-treated WT mice spent significantly more time in the dark chamber relative to saline-treated WT mice (Fig. 3a). Sumatriptan-treated Nav1.9^*−/−*^ mice challenged with SNP showed no signs of photophobia as the animals spent no more time in the dark box than saline-treated WT or Nav1.9^*−/−*^ mice treated likewise (Fig. 3a).

We further quantified the sound sensitivity (phonophobia) of mice following SNP injection by measuring the intensity of sound required to induce the acoustic startle reflex (ASR). We exposed mice to pseudo-random order of 100  ms-long white noise bursts ranging from 60 to 120 dB SPL at 10 kHz. The ASR threshold of saline-treated WT mice injected with SNP was 97 ± 2 dB SPL (*n* = 7), which was significantly different than that of sumatriptan-treated WT mice, which showed a lower ASR threshold of 90 ± 1.5 dB SPL (*n* = 6) (Fig. 3b). Because one decibel equals 10 times the common logarithm of the power ratio, a decrease in ~10 dB SPL in ASR corresponds to a 10-fold increase in sound sensitivity. SNP induced no changes in ASR threshold in sumatriptan-treated Nav1.9^*−/−*^ mice compared to saline-treated Nav1.9^*−/−*^ mice (Fig. 3b). We then examined the prepulse inhibition (PPI) of the ASR. This response provides an operational measure of sensorimotor gating reflecting the sensitization of mice to weak sounds^30^. PPI was measured as the innate reduction of the startle reflex induced by a weak pre-stimulus sound (Fig. 3c). Following SNP injection at day 21, the PPI value was 18.5 ± 2% in saline-treated WT mice but reached 42.4 ± 2.5% in sumatriptan-treated WT mice (Fig. 3c, d), indicating sensitization to sounds. By contrast, the PPI value was not significantly different in saline-treated versus sumatriptan-treated Nav1.9^*−/−*^ mice (Fig. 3c, d).

Together, these data indicate that Nav1.9 is essential to the development of NO-induced symptoms of central sensitization observed in MOH mice.

### Triptan overuse promotes coupling of NO-cGMP to Nav1.9 channels

We investigated the molecular basis that promotes increased sensitivity of MOH mice to NO. Given the difficulty to unambiguously isolate Nav1.9 from Nav1.8 currents when using CsCl-based pipette solution^11,13,14^, retrogradely labeled dural afferent neurons were studied from Nav1.8^*−/−*^ mice. We provided evidence that inactivation of the *Scn10a* gene encoding Nav1.8 did not affect sumatriptan-induced latent sensitization and hypersensitivity to SNP in MOH mice (Supplementary Fig. 3).

**Fig. 4:**
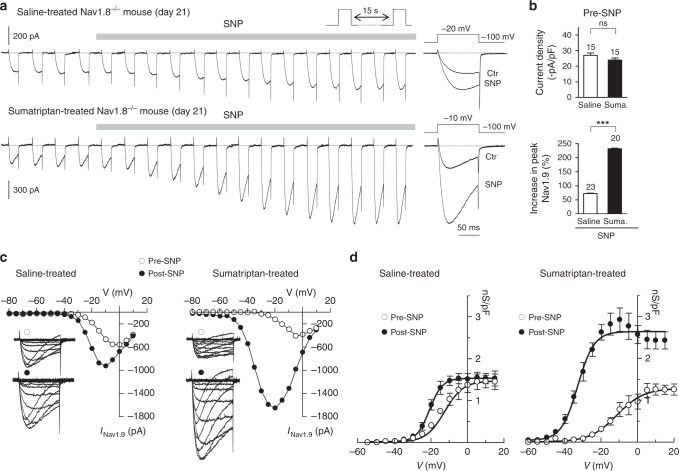
Sumatriptan treatment promotes activation of Nav1.9 by NO. **a** Nav1.9 current exposed to 1 mM SNP in dural afferent neurons from saline-treated (top panel) and sumatriptan-treated (bottom panel) Nav1.8^*−/−*^ mice. CsCl-only-based patch pipette solution. Right-most traces: superimposed Nav1.9 currents before and after SNP application. **b** Nav1.9 current density (top panel) in dural afferent neurons from saline-treated and sumatriptan-treated Nav1.8^*−/−*^ mice. ns, not significant; unpaired *t*-test. Bottom panel: mean increase in Nav1.9 peak current induced by SNP (1 mM) in dural afferent neurons from saline-treated and sumatriptan-treated Nav1.8^*−/−*^ mice. ***p < 0.001; unpaired *t*-test. **c** Nav1.9 *I–V* determined in dural afferent neurons from saline-treated and sumatriptan-treated Nav1.8^*−/−*^ mice before and after SNP exposure. Insets: superimposed Nav1.9 current traces evoked by voltage steps from −80 to +10 mV from a Vh of −100 mV. Note that not all traces are shown for clarity sake. **d** Activation curves for Nav1.9 current determined in DiI^+^-dural neurons before and after SNP application. Boltzmann fits gave *V*_1/2_ values of −14.47 ± 1.3 and −20.8 ± 0.8 mV before and after SNP in saline-treated Nav1.8^*−/−*^ mice (*n* = 11) and of −11.3 ± 1.8 and −32.7 ± 1.02 mV before and after SNP in sumaptriptan-treated Nav1.8^*−/−*^ mice (*n* = 9), respectively. All data collected from neurons cultured at day 21

Nav1.9 current recorded in dural afferent Nav1.8^*−/−*^ neurons cultured at day 21 (i.e. 21 days after pump implantation) was identifiable from its slow activation kinetics and incomplete inactivation, producing ‘persistent’ TTX-resistant Na^+^ currents. Neither the mean Nav1.9 peak current density (Fig. 4b), nor the level of Nav1.9 mRNA expression (at day 21) (Supplementary Fig. 4) was significantly different in TG neurons from saline-treated and sumatriptan-treated mice.

SNP (1 mM) increased Nav1.9 peak current (measured at peak *I–V*) by 232 ± 11% (*n* = 20) in dural afferent neurons from sumatriptan-treated Nav1.8^−/−^ mice, whereas it had little effect (72.7 ± 4%, *n* = 23) in control dural afferent neurons (Fig. 4a, b). Current–voltage relationships determined before and after SNP application in sumatriptan-treated dural neurons showed a 22 mV negative shift in activation *V*_1/2_ value (from −11.3 ± 1.8 to −32.7 ± 1.02 mV). This shift was also associated with a ~2-fold increase in Nav1.9 maximum conductance (from −1.28 to −2.64 nS/pF) (Fig. 4c, d). By contrast, SNP induced a ~−6 mV shift in dural afferent neurons from saline-treated mice (from −14.47 ± 1.3 to −20.8 ± 0.8 mV), which was not associated with change in *G*_max_ (Fig. 4d).

Because the cyclic guanosine monophosphate (cGMP) signal pathway plays an important role in NO signaling, we sought to determine the involvement of the soluble guanylyl cyclase (sGC) in the activation of Nav1.9. Application of methylene blue (100 µM), a sGC inhibitor, abolished the effect of SNP on Nav1.9 in dural afferent neurons from sumatriptan-treated Nav1.8^−/−^ mice (Supplementary Fig. 5). Consistently, the cell-permeable cGMP analog 8-Br-cGMP (1 mM) strongly increased Nav1.9 current and negatively shifted *V*_1/2_ activation by 19 mV (from −13.5 to −32.5 mV) in saline-treated Nav1.8^*−/−*^ mice (Supplementary Fig. 6A–C). These data indicate that NO-cGMP pathway activates Nav1.9 in sumatriptan-treated, but not in saline-treated dural afferent neurons.

### Relief of PKA inhibition causes NO coupling to Nav1.9 in MOH mice

To probe the molecular changes in TG neurons at day 21 from sumatriptan-treated mice, we made qPCR analysis of cGMP-linked signaling molecules. Relative mRNA quantification showed there was a three-fold decrease of the transcript of protein kinase cAMP-activated catalytic subunit alpha (PKA-Cα) but no changes (0.5 < RQ < 2) for the cyclic nucleotide phosphodiesterases 3a, 3b and 5a (PDE3a, PDE3b, and PDE5a), the sGC, the predominant receptor for NO, the Protein Kinase G type I (PKG-I) and the Adenylyl Cyclase type III (AC-III) (Supplementary Fig. 7A). Pre-treatment of 8-Br-cAMP, a membrane permeable cAMP analog, inhibited cGMP-mediated activation of Nav1.9 in dural afferent neurons from saline-treated mice, although it had no effect per se on Nav1.9 (Supplementary Fig. 7B–D), indicating that cAMP can inhibit cGMP coupling to Nav1.9 in TG neurons. Consistently, we found that DiI^+^-dural afferent neurons showed strong staining for the catalytic PKA subunit phospho T197 antibody that recognizes the active form of the PKA protein (Supplementary Fig. 8A, B). In addition, the mean intensity (arbitrary unit) per pixel of PKA subunit phospho T197 staining was significantly (*p* = 0.03, unpaired *t*-test) reduced from 131 ± 4 (*n* = 44 DiI^+^-neurons) in control mice injected with saline solution to 118 ± 5 (*n* = 50 DiI^+^-neurons) in mice treated with sumatriptan (data not shown). Changes in PKA expression in TGs from sumatriptan-treated mice (*n* = 8) were also evaluated by quantifying band intensities on phospho T197 PKA blots and comparing to blot band intensities in saline-treated mice (*n* = 8) as controls. Densitometry analysis of background-subtracted blots from 20 µg of total lysate showed a 22% decrease in phospho T197 PKA expression in sumatriptan-treated mice versus control mice (Supplementary Fig. 8C). This decrease however did not reach significant level due to sample variability (Supplementary Fig. 8D).

Collectively, these data suggest that nitrergic activation of Nav1.9 in sumatriptan-treated mice may result from a decrease in PKA activity in dural afferent neurons and subsequent relief of PKA-mediated inhibition of NO–Nav1.9 coupling.

### Nav1.9-mediated hyperexcitability causes central sensitization

**Fig. 5:**
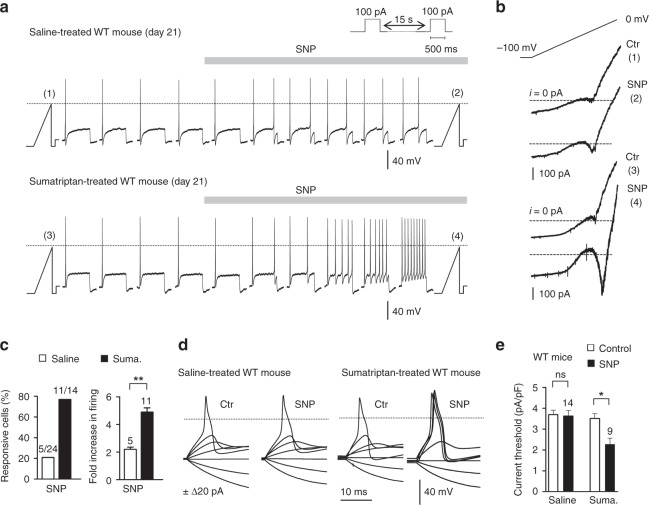
Nav1.9 activation by NO lowers excitability threshold and enhances firing in dural afferent neurons. **a** Effect of SNP (1 mM) on DiI^+^ dural afferent neurons from saline-treated (upper panel) and sumatriptan-treated (bottom panel) WT mice. KCl-based intracellular solution throughout. **b** *I–V* relationships determined using a slow (50 mV/s) voltage ramp command in DiI^+^ dural neurons illustrated in **a** before (1,3) and during (2,4) SNP application. Note the activation of Nav1.9 (inwardly flowing current) by SNP (4). **c** Percentage of DiI^+^ neurons responding to SNP (left panel) and mean change in their firing rate (right panel). Protocol as in **a**. **p < 0.01; Mann–Whitney test. **d** Generation of APs before and after SNP exposure in dural afferent neurons from saline-treated or sumatriptan-treated WT mice. Steady bias currents were used to maintain the neurons at ~−65 mV. **e** Comparison of normalized current threshold for AP before and during SNP application in DiI^+^ dural neurons from saline-treated and sumatriptan-treated WT mice. ns not significant, *p < 0.05 compared to saline-treated WT mice with Wilcoxon matched paired test. All data collected from TG neurons cultured at day 21

Firing activity of retrogradely labeled dural afferent neurons was studied at day 21. SNP-mediated Nav1.9 activation converted phasic discharges into multi-action potential (AP) responses in 78.5% of dural afferent neurons from sumatriptan-treated WT mice (Fig. 5a–c). In addition, SNP reduced by 35% the current threshold for firing in neurons from sumatriptan-treated WT mice (Fig. 5d, e). By contrast, SNP had no significant effects on the firing response and AP current threshold in dural afferent neurons from saline-treated WT mice (Fig. 5c, d). Moreover, SNP caused no changes in excitation or AP current threshold in dural afferent neurons from sumatriptan-treated Nav1.9^*−/−*^ mice (Supplementary Fig. 9).

**Fig. 6:**
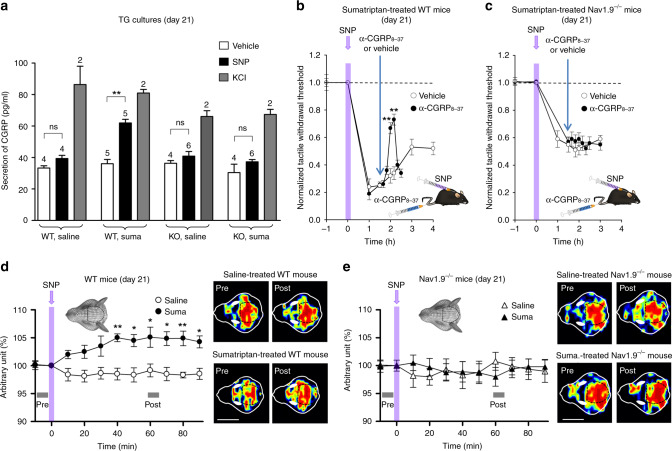
Nav1.9 activation sustains CGRP release, which contributes to central sensitization and meningeal vasodilatation. **a** Effects of SNP (1 mM), HBSS (vehicle), and KCl (40 mM) on CGRP secretion in TG cultures from WT or Nav1.9^*−/−*^ mice treated or not with sumatriptan. The *n* number refers to the number of triplicates. **p < 0.01; Mann–Whitney test. **b**, **c** Effect of intravenous injection of α-CGRP_8–37_ (1 mg/kg) or its vehicle (NaCl 0.9%) on SNP-induced mechanical paw allodynia in sumatriptan-treated WT **b** or Nav1.9^*−/−*^ **c** mice.**p < 0.01 compared to α-CGRP_8–37_ pre-injection with Wilcoxon-matched paired test (*n* = 5 per group). Behavioral tests were carried out at day 21. **d**–**e** SNP-induced meningeal blood flow changes in WT **d** and Nav1.9^*−/−*^ **e** mice, treated or not with sumatriptan. *p < 0.05, **p < 0.01; Mann–Whitney test (*n* = 6 per group). Right panels: representative laser Doppler images taken before and 60 min after SNP injection. The blue color represents low perfusion areas, green and yellow refer to higher perfusion and red shows the highest microperfusion. Scale: 8 mm

We tested whether Nav1.9-mediated hyperexcitability regulates the secretion of CGRP, a key player in headache pathogenesis (Fig. 6a). SNP, at low concentrations, had no significant effects on basal secreted CGRP levels in TG cultures from saline-treated WT mice but enhanced CGRP secretion (+70%) in TG cultures from sumatriptan-treated WT mice. Enhanced secretion of CGRP by SNP was not observed in TG cultures from sumatriptan-treated Nav1.9^*−/−*^ mice (Fig. 6a).

The in vivo consequence of Nav1.9-dependent CGRP secretion was tested on SNP-mediated extracephalic mechanical allodynia at day 21 using the CGRP antagonist α-CGRP_8-37_. Injection of α-CGRP_8-37_ (1 mg/kg), but not the vehicle, reduced SNP-induced extracephalic allodynia in sumatriptan-treated WT mice, indicating that released CGRP contributes to central sensitization (Fig. 6b). Importantly, injection of α-CGRP_8-37_ had no effects on residual SNP-induced allodynia in sumatriptan-treated Nav1.9^−/−^ animals (Fig. 6c), providing further evidence that Nav1.9 activation by SNP is a prerequisite for CGRP release from meningeal nociceptors.

### Nav1.9 mediates vasodilatation and mast cell degranulation

Functional consequences of Nav1.9 activation on meningeal microcirculation was examined at day 21 using a laser Doppler blood perfusion scanner. SNP (0.03 mg/kg), injected through the jugular vein, caused a gradual increase of meningeal blood flow in sumatriptan-treated WT mice. No change was seen in saline-treated WT mice and in sumatriptan-treated Nav1.9^−/−^ mice (Fig. 6d, e). We further tested whether activation of meningeal nociceptors by SNP causes degranulation of dural mast cells (MCs) and pain amplification through a Nav1.9-dependent mechanism. The MC stabilizing agent, sodium cromoglycate (SCG, 10 mg/kg i.p.), injected 30 min prior to SNP, significantly reduced both the intensity and duration of the SNP-induced heightened allodynia in sumatriptan-treated WT mice (Supplementary Fig. 10B) but had no significant effects on SNP-induced basal allodynia in saline-treated WT mice (Supplementary Fig. 10A) and on SNP-induced allodynia in sumatriptan-treated Nav1.9^*−/−*^ mice (Supplementary Fig. 10C).

**Fig. 7:**
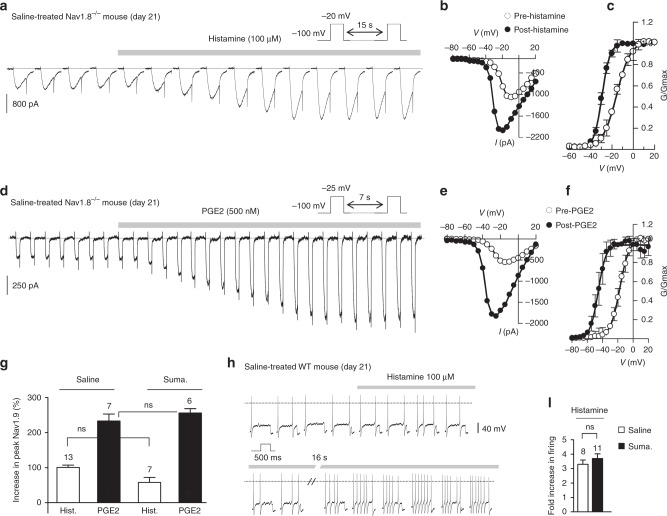
Mast cell mediators activate Nav1.9 in dural afferent neurons. **a** Nav1.9 current challenged with 100 µM histamine in a DiI^+^ dural afferent neuron from a saline-treated Nav1.8^*−/−*^ mouse. CsCl-only-based patch pipette solution. **b** *I–V* relationships from the cell depicted in **a** determined before and after histamine exposure. **c** Averaged activation curve for Nav1.9 determined before and after histamine application in DiI^+^ neurons (*n* = 13). Single Boltzmann fits gave *V*_1/2_ values of −15 ± 0.3 and −29.3 ± 0.15 mV before and after histamine application, respectively. **d** Nav1.9 current challenged with 500 nM PGE2 in a DiI^+^ dural afferent neuron from a saline-treated Nav1.8^*−/−*^ mouse. CsCl-only-based patch pipette solution. **e** *I–V* relationships from the cell depicted in **d** determined before and after PGE2 exposure. **f** Averaged activation curve for Nav1.9 determined before and after PGE2 application in DiI^+^ dural afferent neurons (*n* = 7). Single Boltzmann fits gave *V*_1/2_ values of −17.3 ± 0.6 and −45.6 ± 1.1 mV before and after PGE2 application, respectively. **g** Increase in Nav1.9 current (at peak *I*/*V* as in **b** and **e**) induced by histamine in DiI^+^ neurons from saline-treated or sumatriptan-treated Nav1.8^*−/−*^ mice. ns not significant, Mann–Whitney test. **h** Effects of histamine on a DiI^+^ dural afferent neuron from a WT mouse treated with saline solution. Voltage responses were evoked by depolarizing pulses (+50 pA) applied every 8 s. KCl-based intracellular solution. **i** Mean increase in firing of dural afferent neurons in response to histamine from WT mice treated with sumatriptan or the saline solution. ns not significant, Mann–Whitney test

**Fig. 8:**
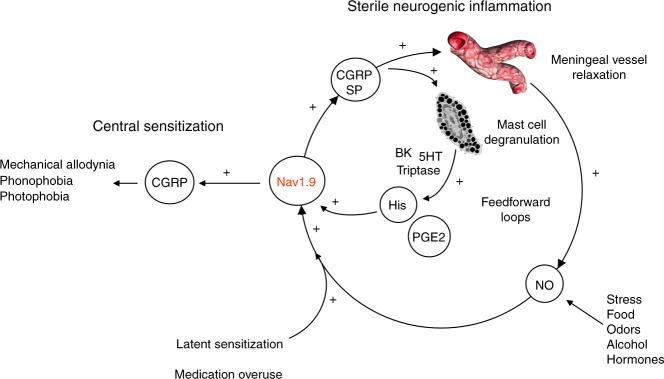
Central role of Nav1.9 in MOH mechanisms. The following scenario summarizes the contribution of Nav1.9 to MOH. NO, which may be released from different sources, activates Nav1.9 channels in dural afferent neurons from chronically treated mice with triptans. Nav1.9 activation by NO increases the excitability of meningeal nociceptors, which sensitizes central structures leading to extracephalic allodynia, photophobia, and phonophobia. Nav1.9-dependent secretion of CGRP in the meninges, possibly in combination with other mediators, causes degranulation of resident mast cells. By releasing histamine and PGE2, MCs retro-excite meningeal nociceptors through Nav1.9 potentiation. Vasoactive peptides also contribute to vascular relaxation that may further facilitate endothelial (and possibly extravascular) NO production. The consequence is a vicious circle that leads to enhanced activation of meningeal nociceptors and maladaptive pain

We finally sought to determine whether MC degranulation contributes to dural afferent terminal excitation through receptor-driven modulation of Nav1.9. Patch clamp recordings showed that the MC mediators histamine and PGE2 are powerful activators of Nav1.9 current in dural afferent neurons (Fig. 7). Superfusion of histamine and PGE2 increased peak Nav1.9 current by 100.5 ± 25% and 233 ± 40%, respectively, in saline-treated Nav1.8 KO mice (Fig. 7a–g). Both mediators caused a substantial leftward shift in the activation curve of Nav1.9 (Fig. 7c, f) and strongly increased the firing rate of dural afferent neurons (Fig. 7h, i). These effects were seen irrespective of the mouse treatment (Fig. 7g). By contrast, CGRP, SP, and neurokinin A, which are potentially released by meningeal terminals, had no detectable effects on Nav1.9 current in dural afferent neurons from either saline-treated or sumatriptan-treated mice (Supplementary Fig. 11A–D). Thus, some MC mediators have the capacity to activate Nav1.9, resulting in a feedforward loop that potentiates neurogenic inflammation and nociceptive transmission. Collectively, these results indicate that Nav1.9 acts as a hub in meningeal nociceptors and contributes to maladaptive nociceptive signal, neurogenic inflammation, meningeal vasodilatation, and mast cell degranulation (Fig. 8).

## Discussion

How chronic exposure to abortive medication leads to MOH remains unclear. Our study suggests that chronic use of triptans induces MOH through abnormal activation of meningeal Nav1.9 by NO. Thus, triptan-overuse headache derives from Nav1.9 activation in the TG system, triggering pain facilitation through central and peripheral sensitization and inflammation in the meninges.

The complex pathophysiology behind MOH is still only partly known. Mechanisms involved may differ from one class of overused drug to another. Previous studies have shown that chronic use of opioids and triptans increases CGRP levels which is well known to be involved in neurogenic inflammation and headache pain^8,31^. Impaired diffuse noxious inhibitory controls^6^ and central sensitization are also seen in MOH patients^4,32^. Many of these phenomena are similar to mechanisms seen in dependence processes^33,34^.

Our approach to modeling MOH symptoms was the quantification of increased sensory sensitivity in response to NO, one of the most common reported trigger for headache and migraine^8,9,35,36^. NO donors reliably trigger headache in normal subjects, but trigger migraine and severe pain in migraineurs^36^, and this condition is accompanied by an increase in blood levels of CGRP, which is directly linked to the severity of headache pain^37–39^. In our study, MOH following NO infusion was evaluated using multiple headache-like responses in each individual animal, including cutaneous facial and extracephalic allodynia as well as aversion to light and noise. Our data show that chronic sumatriptan treatment of mice induces a state of dormant sensitization characterized by sharp, exacerbated sensory responses to NO^8,9,40^.

Clinical and preclinical studies have consistently demonstrated increased excitability of the TG system after medication overuse. TG neuronal hyperexcitability may facilitate the process of peripheral and central sensitization. We show that NO was capable of producing strong activation of dural afferent neurons in MOH mice and that sensory hypersensitivity and MOH-associated symptoms were prevented by deleting Nav1.9 but not Nav1.8. Nav1.9 activation sustains the hyperexcitability of meningeal nociceptors and lowers the threshold response for afferent pain signaling. Importantly, coupling of NO to Nav1.9 was weak under normal conditions, consistent with the observation that deleting Nav1.9 had no impact on SNP-induced (basal) cutaneous allodynia in saline-treated mice. Thus, chronic sumatriptan treatment promotes coupling of NO to Nav1.9 channels in dural afferent neurons, thus lowering the threshold of the animal’s susceptibility to respond to initiating factors of headache. Importantly, we found that hypersensitivity to SNP was greater in MOH female than in male mice, which parallels the sexual dimorphism reported in MOH and migraine in humans.

What then favors Nav1.9 activation by NO/cGMP in sumatriptan-treated mice? PKA-Cα transcripts were found to be down-regulated in TGs from sumatriptan-treated animals at day 21, suggesting that constitutive activity of PKA, in control animals, inhibits NO–Nav1.9 coupling. This is consistent with the observation that cAMP pretreatment inhibited cGMP-mediated activation of Nav1.9 in dural afferent neurons. How chronic stimulation of Gi-coupled 5HT1B/D receptors, which are notably expressed in TG neuron plasma membrane^41^, leads to long-term changes of PKA gene transcription remains to be determined.

Genetic deletion of Nav1.9 prevented SNP-induced meningeal vasodilatation, indicating that Nav1.9-dependent excitation of TG neurons was a prerequisite for this effect. Thus, Nav1.9 activation by SNP not only causes hyperexcitability of nociceptors but also triggers the release of vasoactive neuropeptides in the meninges. In vitro experiments further indicated that NO-induced release of CGRP was prevented by deleting Nav1.9. These data indicate that Nav1.9-dependent release of CGRP, and possibly other neuropeptides including SP, plays a pivotal role in the vasodilatation of meningeal blood vessels. Our data however do not specify whether the vasodilatation contributes to MOH-related symptoms or whether this is a side-phenomenon^42^. Therefore, if vasodilatation of meningeal arteries contributes to the propagation of the cascade of symptoms, it is likely in conjunction with other factors. CGRP is also poised to enhance headache pain by central mechanisms. Consistently, our results show that SNP-induced extracephalic allodynia was transiently alleviated by acute administration of the CGRP antagonist α-CGRP_8–37_ in sumatriptan-treated animals. Because, extracephalic allodynia is a manifestation of central sensitization^43^, these data argue that Nav1.9-dependent CGRP release may also occurs at postsynaptic structures, such as the trigeminal nucleus caudalis, activity of which may sensitize thalamic neurons. These results call for an early use of anti-Nav1.9 drugs that target meningeal nociceptors, before the development of central sensitization.

Our data demonstrate that Nav1.9-mediated release of neuropeptides from meningeal nociceptors could trigger degranulation of dural MCs. CGRP and SP, which are often co-released, are particularly important in this regard as they are known to stimulate MC degranulation in different systems^44^. Histamine, a major amine released from MCs^45^, and PGE_2_ another pro-inflammatory MC mediators^46^, are well-recognized migrainogenic substances known to induce pain upon infusion into human subjects diagnosed with migraine. Our data show that both substances cause a prominent increase of Nav1.9 and promote neuronal hyperexcitability, resulting in a vicious, self-reinforcing cycle of sterile inflammation and nociception (cf Fig. 8). Because MCs express a variety of 5-HT receptors, it is possible that chronic treatment with sumatriptan reinforces the possibility that MCs respond to peptide-induced degranulation. However, the reported effects of 5-HT on MCs have generally been found to be mediated by 5-HT1A and not by 5HT1B/D receptors^47^.

In conclusion, our study identifies NO-induced Nav1.9 channel activation as a triggering mechanism for MOH-related symptoms. The way in which medication overuse transforms episodic migraine into chronic daily headache is still unknown. The picture that emerges from our study is that abnormal sensitivity of meningeal Nav1.9 channels to the migraine trigger NO may cause the headache phase in MOH patients. Activation of Nav1.9 may be a common denominator of overused drugs and migraine triggers. Therefore, the use of Nav1.9 channel inhibitors, in combination with sumatriptan or other headache medications, may represent a new acute and preventive option for migraine treatment.

## Methods

### Animals

This project was approved by the Institutional Review Board of the regional ethic committee. All animals were used in accordance with the European Community guiding in the care and use of animals (2010/63/UE). All efforts were made to minimize the number of animals used and their suffering. Mice (10–12-week-old adult males and females, C57Bl/6J background) used in this study were Nav1.9^*−/−*^, Nav1.8^*−/−*^ (gifts from N.J. Wood, see refs. ^19,23^) and their WT littermates. The Scn11a-GAL reporter mouse was previously described^15^. An IRES/LacZpA cassette followed by a loxP/neo/loxP cassette was inserted into the end of exon 5 of the *Scn11a* gene.

### Infusion of sumatriptan and injection of SNP

Alzet osmotic minipumps (model 1007D, Charles River, France) with a nominal flow rate of 0.5 μl/h for 6 days were used for drug infusion. The minipumps were implanted subcutaneously under anesthesia with isoflurane. The day of the pump implant was considered as day 0. Drugs administered by infusion were sumatriptan (0.6 mg/kg/day, Sigma, St. Louis USA) and its vehicle (NaCl, 0.9%). SNP was injected subcutaneously into the loose skin over the neck at 0.03 mg/kg. Infusion of sumatriptan/saline and injection of SNP/saline were made blind, e.g. the investigator was not aware of the content of the minipump, nor of the nature of the solution (SNP or vehicle) injected on day 21. Animals were randomly assigned to treatment groups.

### Tactile sensory testing

Hind paw mechanical threshold was assessed using von Frey filaments (Bioseb, France) as described^20^. For facial testing, mice were subjected to a von Frey stimulus applied to the forehead surface, repeated three times (at minimum 30 s interval). The head withdrawal tactile sensory threshold was the lowest force to elicit withdrawal in 2 of 3 trials. Data points were normalized to the control sensory threshold values determined just before minipump implantation (i.e. at day 0) or before SNP injection (i.e. at day 21, H0).

### Light aversive test

To evaluate the light-aversive behavior after SNP injection, we used a light–dark test. The light-aversion chamber consisted of two equally sized compartments (10 cm width, 13 cm length, and 13 cm height), one painted white and lacking a top, the other painted black and fully enclosed. A corridor (7.2 cm width, 7.2 cm length, and 13 cm height) connects the two compartments. Mice were acclimatized at least 1 h in their cages in the testing room and were allowed to explore the light-aversion chamber 10 min before testing. The light-aversive behavior was examined 2 h after SNP injection (0.03 mg/kg) and during 30 min. The thermal-neutral fiber-optic source is located in the middle of white box and produces a light intensity of 2600 lx inside the lit area of the white chamber. Mouse transitions were interpreted to be a reflection of light aversion.

### Acoustic startle threshold and prepulse inhibition tests

The Startle Reflex System (Bioseb, France) was used to measure the acoustic sensitivity of mice. The hardware consisted of an isolation cabinet that minimizes the effects of extraneous noise and vibrations. The startle chamber is situated in the center of the isolation cabinet (250 (*W*) × 250 (*D*) × 250(*H*) mm) and a restrainer (90 × 30 mm) was used to restrain minimally the animal during testing. Mice were acclimatized in the restrainer for 5 min per day, one week before the testing session. Mice were also acclimatized in their cages in the testing room, 2 h before the testing session. Acoustic stimuli for behavioral tests were based on previously published studies defining criteria applicable to mice at around 2 months of age. The waveforms generated by the mouse’s movement during the startle response were analyzed using the Packwin software (Panlab, Inc., Harvard apparatus).

Two hours after SNP injection, the mouse was acclimatized for 5 min in the presence of a background noise level of 60 dB SPL. The mouse was then exposed to random trials of white noise bursts ranging from 60 to 120 dB SPL at 10 kHz, frequency that falls within the most sensitive region of the mouse audiogram. Each sound intensity was presented three times. The ASR threshold was taken as the minimum intensity required to elicit a response in two out of third trials. The PPI is a reliable, robust quantitative phenotype that is useful for probing sensory functions. Testing consisted of a series of 115 dB SPL-test pulses immediately preceded or not by a prepulse of 70 dB SPL at 10 kHz. PPI was calculated as follows: PPI (%) = (1–(startle response with prepulse)/(startle response without prepulse)) × 100.

### Cultures of TG neurons

Mice were anesthetized at day 21 with isoflurane and killed by decapitation. TGs were dissected out and freed from their connective tissue sheaths. TG neurons were incubated in enzyme solution containing 2 mg/ml of collagenase IA (Sigma) for 45 min at 37 °C. The tissue was washed several times and triturated in Hanks’ balanced salt solution. The resulting suspension was filtered (70 µm filters) and centrifuged (800×*g* for 5 min) and plated on poly-l-lysine/laminin (0.05 and 0.01 mg/ml, respectively) coated Nunclon dishes. Culture medium was Dulbecco’s modified Eagle’s medium supplemented with 10% heat-inactivated FCS, 100 U/ml penicillin–streptomycin, 2 mM l-glutamine, 25 ng/ml nerve growth factor (NGF), and 2 ng/ml glial-derived neurotrophic factor (GDNF).

### Patch clamp recordings

Patch pipettes had resistances of 2 MΩ. Voltage clamp recordings of Na^+^ currents used the following intracellular solutions: (CsF-containing, in mM), 30 CsF, 100 CsCl, 10 HEPES, 10 EGTA, 8 NaCl, 1 MgCl_2_, 1 CaCl_2_, 4 MgATP, 0.4 Na_2_GTP (pH 7.35, 300 mOsm/l); (CsCl-based, in mM), 130 CsCl, 10 HEPES, 10 EGTA, 8 NaCl, 1 MgCl_2_, 1 CaCl_2_, 4 MgATP, 0.4 Na_2_GTP (pH 7.35, 300 mOsm/l). The extracellular solution contained (in mM): 60 NaCl, 110 sucrose, 3 KCl, 1 MgCl_2_, 10 HEPES, 2.5 CaCl_2_, 10 glucose, 10 TEA–Cl, 0.0005 TTX, 1 amiloride, 0.05 La^3+^ (pH 7.4, 305 mOsm/l). For current clamp recording, the intracellular solution (KCl-based) consisted of (mM): 115 KCl, 10 HEPES, 10 EGTA, 8 NaCl, 1 MgCl_2_, 1 CaCl_2_, 4 MgATP, 0.4 Na_2_GTP. The extracellular solution consisted of (in mM) 131 NaCl, 3 KCl, 1 MgCl_2_, 10 HEPES, 2.5 CaCl_2_, 10 glucose (pH 7.4, 305 mOsm/l). All chemicals were from Sigma-Aldrich, except TTX (Alomone Labs).

PCLAMP 9.2 (Axon Instruments Inc.) and PRISM 4.0 (GraphPad) software suites were used to perform linear and nonlinear fitting of data. Conductance–voltage curves were calculated from the peak current according to the equation *G* = *I*/(*V*−*E*_rev_), where *V* is the test pulse potential and *E*_rev_ the reversal potential calculated according to the Nernst equation. The activation curve (*G*−*V*) was fitted using the Boltzmann function: *G*/*G*_max_ = 1/(1 + exp[(*V*_1/2_−*V*)/*k*]), where *G*/*G*_max_ is the normalized conductance, *V*_1/2_ is the potential of half-maximum channel activation, and *k* is the steepness factor.

### Tracer application onto the dura

Mice were anesthetized with isoflurane. Throughout surgery, the core temperature of the mouse was monitored and maintained by an homeothermic blanket system for rodents. Two small cranial windows were made in parietal bones and the retrograde nerve tracer DiI (DiI tissue labeling paste, Invitrogen) was applied onto the dura. The bone flaps were then replaced after the procedure with bone wax in order to prevent tracer spreading. Animals were euthanized for TG extraction 2 days after DiI application.

### Tissue preparation, immunostaining, and confocal imaging

For Nav1.9 immunostaining, the tissues were cryoprotected in PBS containing 4% sucrose during 30 min and then incubated for at least 1 h in PBS plus 20% sucrose at 4 °C. The TGs were frozen in OCT embedding matrix bathed in chilled isopentane. The TGs were then sagitally cryosectioned at 14–18 µm, transferred to SuperFrostPlus slides and stored at −80 °C until processed. Whole mount dura maters were transferred to SuperFrostPlus slides, frozen on dry ice and stored at −80 °C until processed. Primary antibodies used and dilutions were as follows: anti-peripherin 1/400 (mouse monoclonal, Millipore, Temecula, CA); anti-NF200 1/400 (chicken polyclonal, Aves Labs, Tigard, OR); anti-CD31 (1/400, rat polyclonal, BD Biosciences, Belgium); anti-Nav1.9 L23, (1/100, rabbit polyclonal)^10^; and anti-CGRP (1/300, goat polyclonal, AbCam).

For CD31 immunostaining, the tissues were fixed for 30 min at room temperature (RT) in Antigenfix (Microm Microtech, France) before being cryoprotected and frozen as described above. Slides with fresh frozen tissues or fixed with Antigenfix tissues were thawed at RT and then incubated for 90 min at RT in blocking solution containing 3% BSA and 0.1% Triton X-100. Primary antibodies were diluted in PBS containing 3% BSA and applied to tissues to be incubated overnight at 4 °C in sealed humidified chambers.

For CGRP immunostaining, the tissues were fixed in 4% PFA in PBS for 3 h at RT then cryoprotected in PBS containing 25% sucrose overnight at 4 °C. The tissues were transferred to SuperFrostPlus slides, frozen and stored at −80 °C until processed. Slides with PAF-fixed tissues were thawed at RT and then incubated for 1 h30 min at RT in blocking solution containing 5% fish gelatin and 0.2% Triton X-100. CGRP antibodies were diluted in PBS containing 5% fish gelatin and applied to tissues to be incubated overnight at RT in sealed humidified chambers.

After incubation with the primary antibodies, fixed and fresh frozen tissues were identically proceeded. Tissues were washed three times for 5 min in PBS and incubated for 45 min at RT with secondary antibodies diluted in blocking buffer. Secondary antibodies were: Alexa Fluor 647-conjugated donkey anti-mouse (1/400, Life Technologies), TRITC-conjugated donkey anti-rabbit (1/400, Jackson ImmunoResearch, Suffolk, UK), TRITC-conjugated donkey anti-rat (1/100, Jackson ImmunoResearch), Alexa Fluor 488-conjugated donkey anti-goat (1/200, Life Technologies). After six 5 min-washes in PBS, sections were mounted in Mowiol (Sigma-Aldrich). Images were acquired using a LSM 780 laser-scanning confocal microscope (Zeiss), initially processed using ZEN software (Zeiss) and later exported into Adobe Photoshop (Adobe Systems, San Jose, CA) for final processing.

### Staining for beta-galactosidase enzyme activity

Evaluation of β-gal enzymatic activity was performed on transgenic mice that express β-gal at the *Scn11a* loci. Once dissected out, the TGs were slightly fixed 15 min in PBS containing 2% PFA, 0.2% glutaraldehyde, and 2 mM MgCl_2_. The tissues were washed with two changes of PBS containing 2 mM MgCl_2_ for 5 and 8 min. The staining is achieved with an overnight incubation of TGs in PBS added with 4 mM Ferrocyanide, 4 mM Ferricyanide, 2 mM MgCl_2_, 1% Tween-20, and 0.2 mg/ml X-gal at 37 °C. The TGs were finally post-fixed for 15 min in PBS containing 2% PFA, 0.2% glutaraldehyde, and 2 mM MgCl_2_, and washed four times in PBS. The TGs were cryoprotected in PBS containing 4% sucrose for 30 min and then incubated overnight in PBS plus 20% sucrose at 4 °C. The TGs were then frozen in OCT embedding matrix (Cellpath, Hemel Hempstead, UK) bathed in chilled isopentane. TGs were then sagitally cryosectioned at 14–18 µm, transferred to SuperFrostPlus slides (Fisher Scientific, Houston, TX), dried at RT and mounted in Mowiol (Sigma-Aldrich).

### CGRP staining in TGs from *Scn11a*-GAL reporter mice

β-gal enzymatic activity in TGs from *Scn11a*-GAL reporter transgenic mice was revealed as above, except that glutaraldehyde was omitted from the fixative solution. Once achieved, TGs were post-fixed for 2 h in PBS containing 2% PFA and 2 mM MgCl_2_, washed four times in PBS and then cryoprotected in PBS containing 25% sucrose overnight. TGs were then frozen in OCT embedding matrix bathed in chilled isopentane and sagitally cryosectioned at 14–18 µm, transferred to SuperFrostPlus slides, dried at RT and stored at −80 °C. Slides with TGs were thawed for 15 min at RT and then incubated for 1 h 30 min at RT in blocking solution containing 3% BSA and 0.1% Tx-100. CGRP antibody (rabbit polyclonal #PC205L, Millipore) were diluted at 1/200 in blocking buffer and applied to tissues to be incubated overnight at 4 °C in sealed humidified chambers. Tissues were washed three times for 10 min in PBS and incubated 40 min at RT in PBS containing 3% BSA added with secondary antibodies Alexa Fluor 488-conjugated donkey anti-rabbit (1/200, Life Technologies). After successive 5 min washes in PBS, sections were mounted in Mowiol (Sigma-Aldrich). Images were acquired using a conventional fluorescence microscope (Zeiss Axio-observer) with constant acquisition settings.

### Quantitation of anti-PKA immunostaining

The TG ganglia were carefully dissected out from NaCl-treated and sumatriptan-treated mice whose dura were DiI-stained (see above). The TGs were fixed in 4% PFA in PBS for 2 h 30 min at 4 °C then cryoprotected in PBS containing 25% sucrose overnight at 4 °C. The TGs were then frozen in OCT embedding matrix bathed in chilled isopentane and stored at −80 °C until processed. The day before PKA staining, the TGs were sagitally cryosectioned at 14 µm, transferred to SuperFrostPlus slides and stored at −80 °C. Slides with PAF TGs were thawed 15 min at RT and then incubated for 30 min at RT in blocking solution containing 3% fish gelatin and 0.05% saponin. PKA antibodies (rabbit polyclonal #ab75991, AbCam) were diluted at 1/200 in blocking buffer and applied to tissues to be incubated overnight at 4 °C in sealed humidified chambers. Tissues were washed three times for 10 min in PBS and incubated for 3 h at RT in PBS containing 3% fish gelatin added with secondary antibodies Alexa Fluor 488-conjugated donkey anti-rabbit (1/200, Life Technologies). After four 5 min washes in PBS, sections were stained with DAPI, washed once in PBS and mounted in Mowiol (Sigma-Aldrich).

In order to compare the fluorescence intensities of PKA immunostaining between NaCl-treated and sumatriptan-treated DiI-positive TG neurons, images were acquired using a LSM 780 laser-scanning confocal microscope (Zeiss) using constant acquisition settings. ImageJ software was used to measure intensities of fluorescence on planar projections of confocal raw images spanning 6 µm. Elliptical regions of interest encompassing the soma of each DiI-positive neuron were defined in the DAPI image to exclude staining of the nucleus. The measurements were repeated in an adjacent area out of the tissue and the resulting background intensities were subtracted from the soma fluorescence signal. Results are expressed as mean grey value intensity per pixel meaning the sum of grey values of all pixels in the ROI divided by the number of pixels.

### PKA western blot analysis

TGs were collected at 21 days after sumatriptan or saline infusion (T0), scrapped in TE buffer (66 mM Tris pH 6.8, 2% SDS, 10% glycerol, 0.1 M DTT, and antiprotease 2×) and sonicated 3 × 5 s. The protein concentration was analyzed by spectrophotometer (Nanodrop); concentrations were normalized to 800 µg/ml in TE and bromophenol blue (30 mM). Electrophoresis was performed via SDS–PAGE using bis/tris polyacrylamide gels 4–12% (Thermo Scientific). After blotting, nitrocellulose membranes were stained with ponceau red. For immunostaining, membranes were washed in TBST, blocked in blocking solution (5% milk in TBST) and incubated with anti-PKA (rabbit monoclonal, 1:5000, Abcam, ab75991) and anti-GAPDH (mouse monoclonal, 1:5000, Millipore, MAB374) antibodies overnight at 4 °C. The next day, the membranes were washed three times in TBST, incubated with secondary polyclonal-HRP-conjugated goat anti-rabbit (1:2000, Biorad, 1706515) or anti-mouse (1:2000, polyclonal, Biorad, 1706516) antibody for 1 h at RT and washed again three times in TBST. Afterward, immunoreactivity was visualized using ECL Western blotting substrate (Roche) and densitometrically quantified using a Gbox Analyzer (Syngene). Reported mean grey values were determined with ImageJ.

### CGRP secretion

TGs were dissected out at day 21 and cultured for 1 DIV. Cells were plated in flat bottom plates at a concentration of 15,000 cells per well. The supernatant of cultured TG neurons was removed for CGRP detection after 30 min incubation with KCl (40 mM), SNP (1 mM), or the vehicle (0.9% NaCl). CGRP detection was made using an enzyme-linked immunoabsorbent assay kit (Spibio, Berlin Pharma, France). Samples were run in triplicate.

### Laser Doppler blood perfusion scanning

Meningeal microcirculation was determined in isoflurane-anesthesized mice with a laser Doppler blood perfusion scanner (Periscan PIM-III, Perimed) through the closed cranium after removing the covering skin and the periosteum. Light particles that hit moving blood cells undergo a change in wavelength/frequency, while light particles which encounter static structures return unchanged. The scattered light that returns to the tissue surface is registered by a photodetector. The perfusion can be calculated since the magnitude and frequency distribution of the Doppler shifted light are directly related to the number and velocity of blood cells, but unrelated to their direction of movement. Although the measured parameter is flux, this signal is then processed to extract information about the microcirculatory blood flow. After a control period (10 min), SNP (0.03 mg/kg) was injected into the jugular vein catheter and the observation period lasted for 90 min.

### Quantitative real-time RT-PCR

Total RNA was extracted from TG tissue using the RNeasy^®^Plus minikit (Qiagen) and assessed on a NanoDrop ND-2000 spectrophotometer (Thermoscientific) for concentration (*A*_260_) and purity by OD ratios (*A*_260_/*A*_280_, ranging between 2.0 and 2.2). cDNA was synthesized by using 1.2 µg total RNA from each sample with oligo-dT primers and Superscript III® (Invitrogen) in 20 µl reactions according to the manufacturer’s instructions. To assess the specificity of these reactions, no reverse transcriptase and no template controls were also generated. cDNA was stored at −20 °C before PCR detection. RT products were diluted and amplified in 12 µl reactions using Kapa Sybr® Fast qPCR kits (Kapa Biosystems). GAPDH was selected as reference gene for normalization of the qPCR results. qPCR reactions were run in duplicate on a Applied Biosystems 7500 Fast Real-time PCR thermocycler using MicroAmp® Fast 96-well reaction plates. Thermocycling parameters were 40 cycles of 95 °C for 3 s for denaturation and 60 °C for 30 s for annealing and extension. Parameters and reaction conditions were identical for all sets of primers. To compare transcript levels between saline and sumatriptan TG samples, the relative quantification (RQ) method was used. First, the difference (∆Ct) between the cycle threshold (Ct) values of the target gene and the *GAPDH* gene was calculated. Then, the difference (∆∆Ct) between the normalized values with or without sumatriptan was calculated: ∆∆Ct = ∆Ct_sumatriptan_−∆Ct_saline_. Finally, to determine the ratio of expression levels in sumatriptan samples versus saline samples, we used the RQ formula as follows RQ = 2 ^−∆∆Ct^. Applied Biosystems 7500 Software v 2.0.6 was used for analysis. Primers used were described in Table 1.

**Table 1:** Primers used in q-PCR analysis

Gene	Accession no.	Targeted sequence (5′−3′)
Nav1.7	XM_006499033	f-CCTTGGCCCCATTAAATCTCT r-TGCTCCTATGAGTGCGTTGAC
Nav1.8	XM_006511991	f-TTGACACAACCTCGCTCTATTCC r-ATTTCACCCTGGGTCTTCTCTCA
Nav1.9	AF118044	f-CCCTTGTGAGTCTCGCTGAC r-GGAGTGGCCGATGATCTTAAT
5HT1B	NM_010482.1	f-TCGTTGCCACCCTTCTTCTG r-CGTGGTCGGTGTTCACAAAG
5HT1D	NM_008309.4	f-GGGTCAATTCATCAAGAACACA r-GCTTGGAAGCTCTGAGGTGT
PDE3a	NM_018779	f-AATGGGACCACAAGAGAGGG r-TTCACTCTGGGCTTGTGGAT
PDE3b	NM_011055	f-AAACGATCGCCTCTTGGTCT r-CCCAGGGTTGCTTCTTCATC
PDE5a	NM_153422	f-GGAAATGGTGGGACCTTCACT r-AAGAACAATACCACAGAATGCCA
GC	NM_021896	f-TGTTCACCTCTGCAGGTCAT r-CCACACAATATGCATCCCCG
PKG2	NM_008926.4	f-CGGAAGAGTGGAGCTTGTTA r-AGCCATCGGCATCCAGAATTA
AC3	NM_138305	f- ACATGATGCCCACGATGATA r-CAGCAGGATGAGCTGGAAG
PKAcA	BC054834.1	f-GCAAAGGCTACAACAAGGC r-ATGGCAATCCAGTCAGTCG
GADPH	NM_008084	f-GCAAATTCAACGGCACA r-CACCAGTAGACTCCACGAC

### Statistical analysis

All values are shown as mean ± standard error of the mean (SEM) and *n* represents the number of animals or cells examined. Except for some behavioral experiments, no statistical methods were used to pre-determine sample sizes but our sample sizes are similar to those reported in previous publications. Assessment of normality for sample >14 was tested using the Kolmogorov–Smirnov or the D’Agostino–Pearson Omnibus K2 normality test. Tests for differences between two normally distributed populations were performed using two-tailed *t*-test. Small sample size lacks power to test normality, therefore we typically used non-parametric Mann–Whitney test and Wilcoxon’s test to test for differences between two populations in small samples (*n* ≤ 15). Two-way (repeated measures) ANOVA followed by Student–Newman–Keuls multiple comparison procedure was used for experiments with multiple groups and two dependent variables. Figure legends specify which test was used for specific experiments. Significant levels were set at *p* ≤ 0.05. Analysis used a combination of Clampfit 9.2 (Molecular Devices), Origin 7.0 (OriginLab), and PRISM 7.0 (GraphPad) softwares.

### Reporting summary

Further information on research design is available in the Nature Research Reporting Summary linked to this article.

## Supplementary information

Supplementary Information

Peer Review

Reporting Summary

## Data Availability

The authors declare that all the data supporting the findings of this study are included in the article (or in the Supplementary material) and available from the corresponding author (P.D.).
