# Author Correction: Maladaptive activation of Nav1.9 channels by nitric oxide causes triptan-induced medication overuse headache

**DOI:** 10.1038/s41467-021-26676-z

**Published:** 2021-11-23

**Authors:** Caroline Bonnet, Jizhe Hao, Nancy Osorio, Anne Donnet, Virginie Penalba, Jérôme Ruel, Patrick Delmas

**Affiliations:** 1 Aix-Marseille-Université, CNRS, Laboratoire de Neurosciences Cognitives, UMR 7291, CS8011, Bd Pierre Dramard, 13344 Marseille, France; 2 Centre d’évaluation et de traitement de la douleur, Hôpital de la Timone, Marseille, France

**Keywords:** Diseases, Neurological disorders, Headache, Migraine

Correction to: *Nature Communications* 10.1038/s41467-019-12197-3, published online 18 September 2019.

The original version of the Article contained an error in Fig. 1e, in which the image of Dye-I positive trigeminal neurons in culture was a composite of two fields of the same petri dish, but this separation was not made clear. In the corrected version of Fig. 1e, the two fields are separated by a white box. The figure legend for Fig. 1e has also been updated to include the following clarifying sentence “This is a composite image of two different fields of the same culture dish indicated by a white box” after the sentence “Left panel: TG neurons cultured 2 days after DiI application through a cranial window in the parietal bone.”

In addition, the original version of Fig. 1e showed an arrow adjacent to the Dye-I labelled neuron. However, this image was representative, and the neuron was not used for recording. The arrow has been removed in the corrected version of Fig. 1e.

