# Supplementary material for: Maladaptive activation of Nav1.9 channels by nitric oxide causes triptan-induced medication overuse headache
Source: Nat Commun. 2019 Sep 18;10:4253. doi: 10.1038/s41467-019-12197-3 (PMC6751217; doi:10.1038/s41467-019-12197-3)
Supplement: Supplementary file 1 — Supplementary Information [file 41467_2019_12197_MOESM1_ESM.pdf]

Maladaptive activation of Nav1.9 channels by Nitric Oxide causes Triptan-induced Medication Overuse Headache

Bonnet et al.,

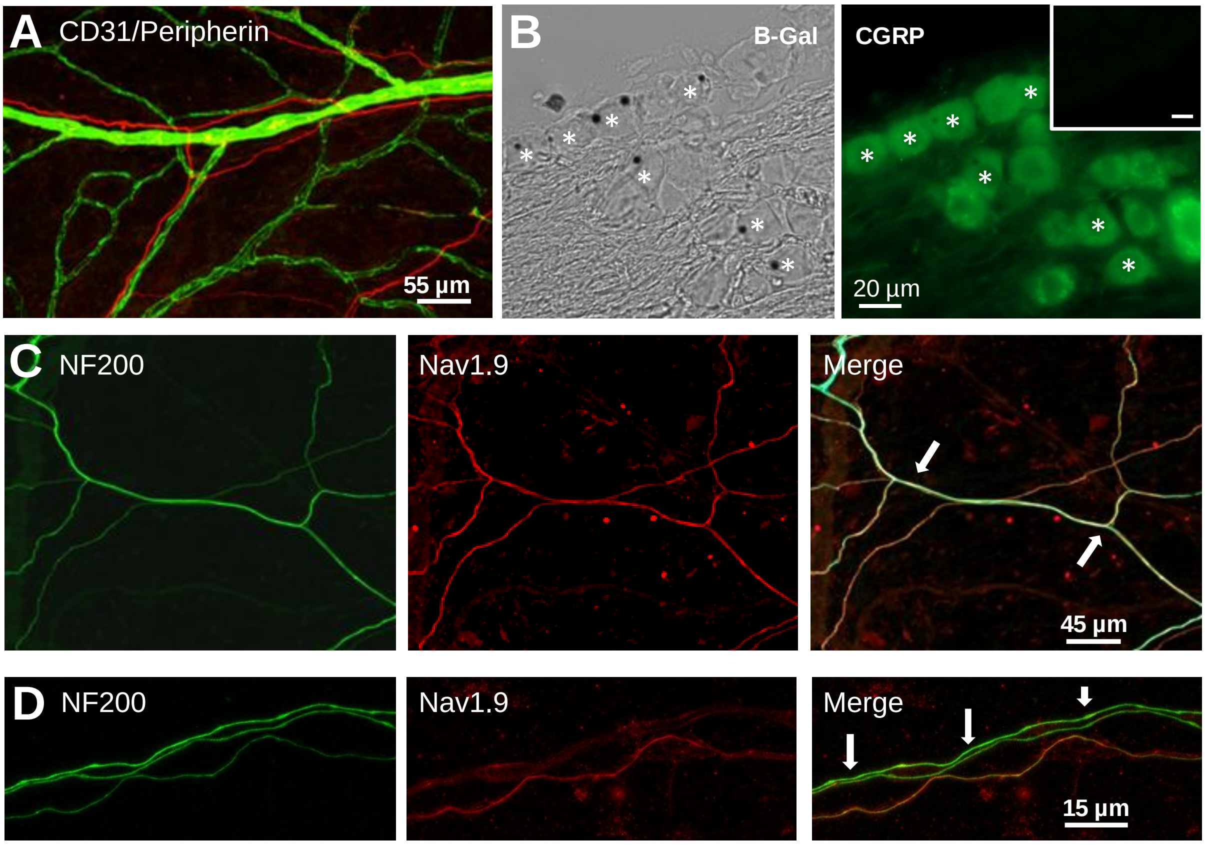

Supplementary Figure 1. Nav1.9 is expressed in most CGRP-positive TG neurons and in few meningeal A-type nerve fibers

(A) Immunostaining for peripherin and CD31 in whole mount of mouse dura mater, showing features of apposition of peripherin-positive fibers with meningeal arteries. Images are projections of 17 consecutive optical sections spanning 18 µm.

(B) TG cryosections from a *Scn11a*-GAL reporter transgenic mouse was labeled for CGRP (green). β-Gal enzymatic activity is revealed by black dots located in the soma of TG neurons. The quasi-totality of examined *Scn11a* expressing-TG neurons shows CGRP labelling (asterisks, n = 63/64). Inset: immunostaining omitting the anti-CGRP antibody. Scale, 20 µm.

(C) Example of co-localisation of Nav1.9 and NF200 in nerve fibers from whole-mount of mouse dura mater. Images are projections of 15 consecutive optical sections spanning 20 µm. The rightmost panel shows the merged image with co-localization (yellow) in some fibers (arrows).

(D) Example of absence of co-localization of Nav1.9 and NF200 in nerve fibers from whole-mount of mouse dura mater. Images are projections of 15 consecutive optical sections spanning 20 µm. The rightmost panel shows the merged image with no co-localization (green) in some fibers (arrows).

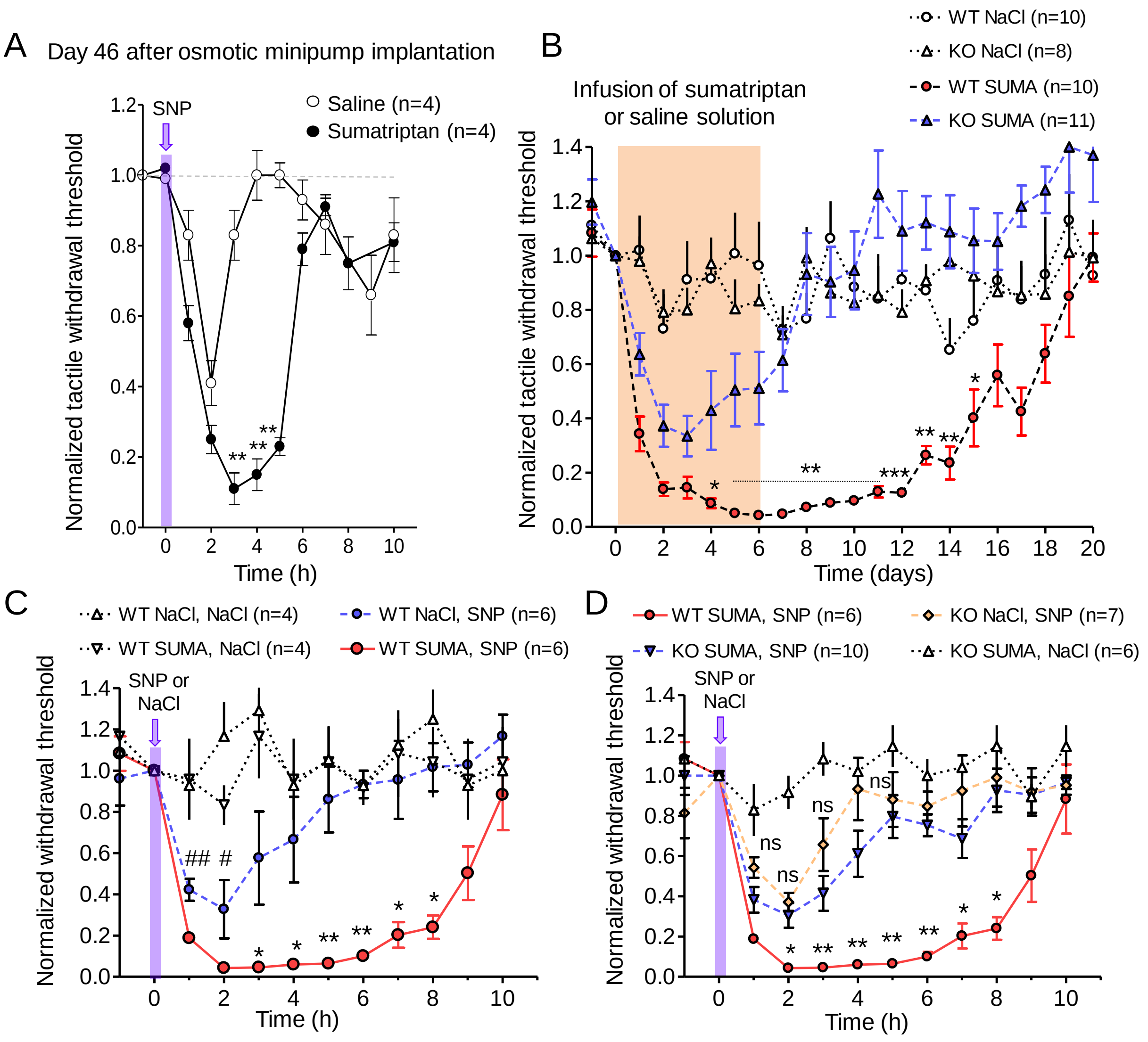

Supplementary Figure 2. Sumatriptan causes long-lasting hypersensitivity to NO in male mice and causes NO-induced generalized allodynia in female mice through Nav1.9-dependent pathways

(A) Changes in hind paw withdrawal mechanical thresholds induced by injection of SNP (0.03 mg/kg) in WT male mice pre-treated with sumatriptan (filled circles) or the saline solution (open circles), 46 days after minipump implantation. **p<0.01 with Mann-Whitney test.

(B) Hind paw withdrawal responses of sumatriptan-treated Nav1.9$^{-/-}$ female mice (n=11) compared with sumatriptan-treated WT female littermates (n=10). Note the strong reduction of mechanical hypersensitivity in sumatriptan-treated Nav1.9$^{-/-}$ female mice compared to WT female littermates. Infusion of saline solution (vehicle, 0.9% NaCl) in both genotypes does not decrease withdrawal thresholds to tactile stimuli applied to the hind paws. Sumatriptan infusion : 0.6 mg/kg/day for 6 days. *p<0.05, **p<0.01, ***p<0.001 compared to KO SUMA with Mann-Whitney non-parametric test.

(C) Comparison of SNP-induced changes in hind paw withdrawal thresholds in sumatriptan-treated WT female mice (red symbols, n=6) and saline-treated WT female mice (blue symbols, n=6). Note that saline injection causes no hypersensitivity in either sumatriptan-treated or saline-treated WT female mice (downward and upward triangles, respectively). All tests were made at day 21. *p<0.05, **p<0.01 compared to WT NaCl, SNP with two-way ANOVA followed by Student-Newman-Keuls test. # p<0.05, ## p<0.01 compared to WT NaCl, NaCl with two-way ANOVA followed by Student-Newman-Keuls test.

(D) Comparison of SNP-induced changes in hind paw withdrawal thresholds in sumatriptan-treated Nav1.9$^{-/-}$ female mice (blue symbols, n=10), saline-treated Nav1.9$^{-/-}$ female mice (orange lozenges, n=7) and sumatriptan-treated WT female mice (red symbols, n= 6, same data as in C). Note the strong reduction of SNP-induced mechanical allodynia in sumatriptan-treated Nav1.9$^{-/-}$ female mice. *p<0.05, **p<0.01 compared to KO SUMA, SNP with two-way ANOVA followed by Student-Newman-Keuls test. ns, not significant compared to KO SUMA, SNP with two-way ANOVA followed by Student-Newman-Keuls test. All tests were made at day 21.

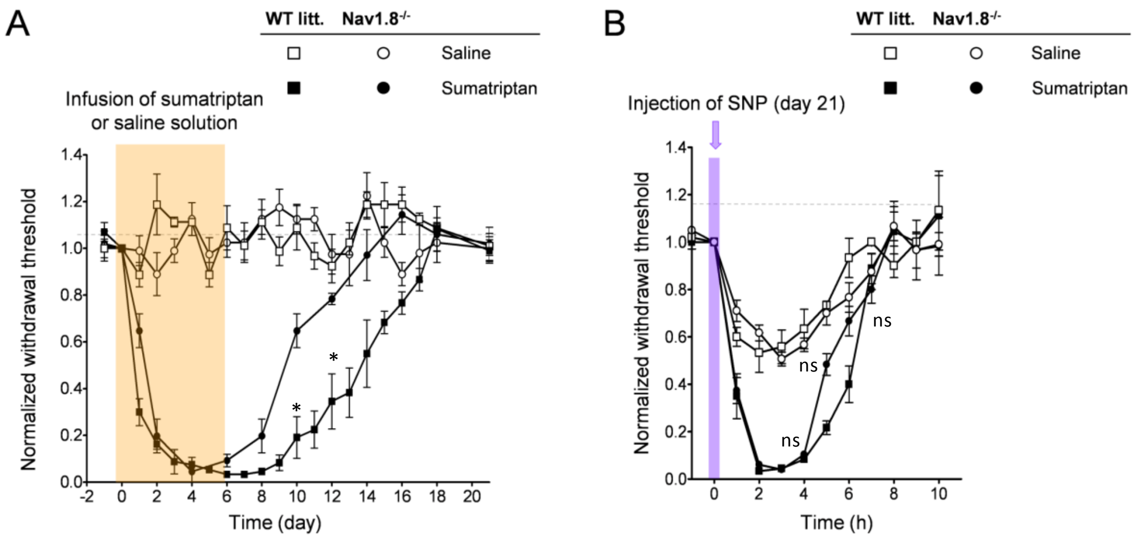

Supplementary Figure 3. SNP induces heightened tactile allodynia in sumatriptan-treated Nav1.8$^{-/-}$ mice

(A) Infusion of sumatriptan, but not saline solution (NaCl 0.9%), decreases withdrawal thresholds to tactile stimuli applied to the hind paws of Nav1.8$^{-/-}$ mice (n=8) and WT littermates (n=8). *p<0.05, compared to Nav1.8 KO SUMA with Mann-Whitney non-parametric test.

(B) Changes in withdrawal mechanical thresholds induced by SNP injection (0.03 mg/kg) at day 21 in Nav1.8$^{-/-}$ mice and WT littermates pre-treated or not with sumatriptan. n=8 mice per group. Note that Nav1.8 deletion had no significant effects on SNP-induced heightened allodynia in sumatriptan-treated mice. ns, not significant compared to WT SUMA, SNP with Mann-Whitney non-parametric test.

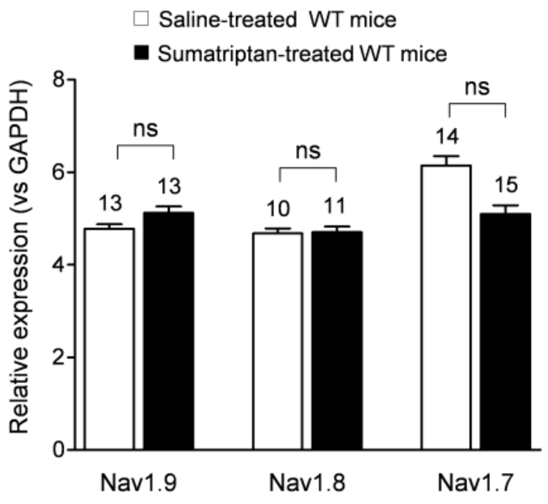

Supplementary Figure 4. SNP-induced heightened tactile allodynia in sumatriptan-treated mice was not associated with changes in transcriptional expression of Nav1.9

Histogram showing the expression of Nav1.7, Nav1.8 and Nav1.9 transcripts relative to GAPDH as assessed by qPCR. TGs were dissected out at day 21 in mice treated with saline solution or sumatriptan. n indicates the number of mice. ns, not significant compared to saline-treated WT mice with Mann-Whitney non-parametric test.

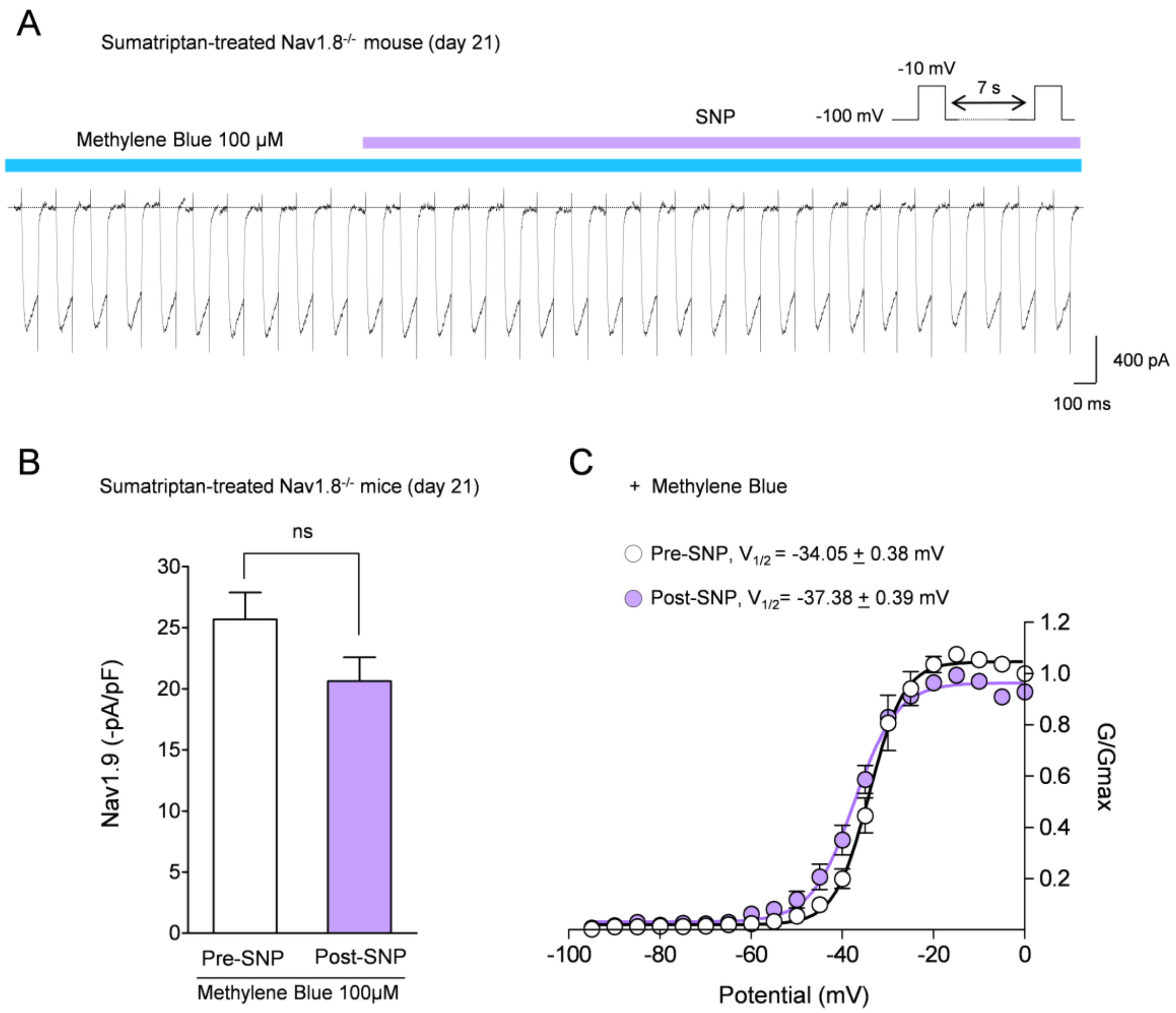

Supplementary Figure 5. Guanylyl cyclase inhibition prevents SNP-induced activation of Nav1.9 in dural afferent neurons from sumatriptan-treated mice

(A) Effects of the superfusion of methylene blue (100 µM), a sGC inhibitor, on SNP (1 mM) activation of Nav1.9 current recorded in a dural afferent neuron from a sumatriptan-treated Nav1.8$^{-/-}$ mouse. Currents were evoked from a holding potential of -100 to -10 mV every 7 s. CsCl-only-based patch pipette solution. TGs were cultured at day 21.

(B) Histogram showing change in mean Nav1.9 current density before and after application of SNP in dural afferent neurons pre-treated or not with methylene blue (100 µM). Recordings were made from sumatriptan-treated Nav1.8$^{-/-}$ TGs cultured at day 21 (n=8). ns, not significant with Wilcoxon matched paired test.

(C) Averaged activation curves for Nav1.9 determined before and after SNP application in dural afferent neurons (n=8) pretreated with methylene blue. Single Boltzmann fits show no significant changes in activation $V_{1/2}$ values.

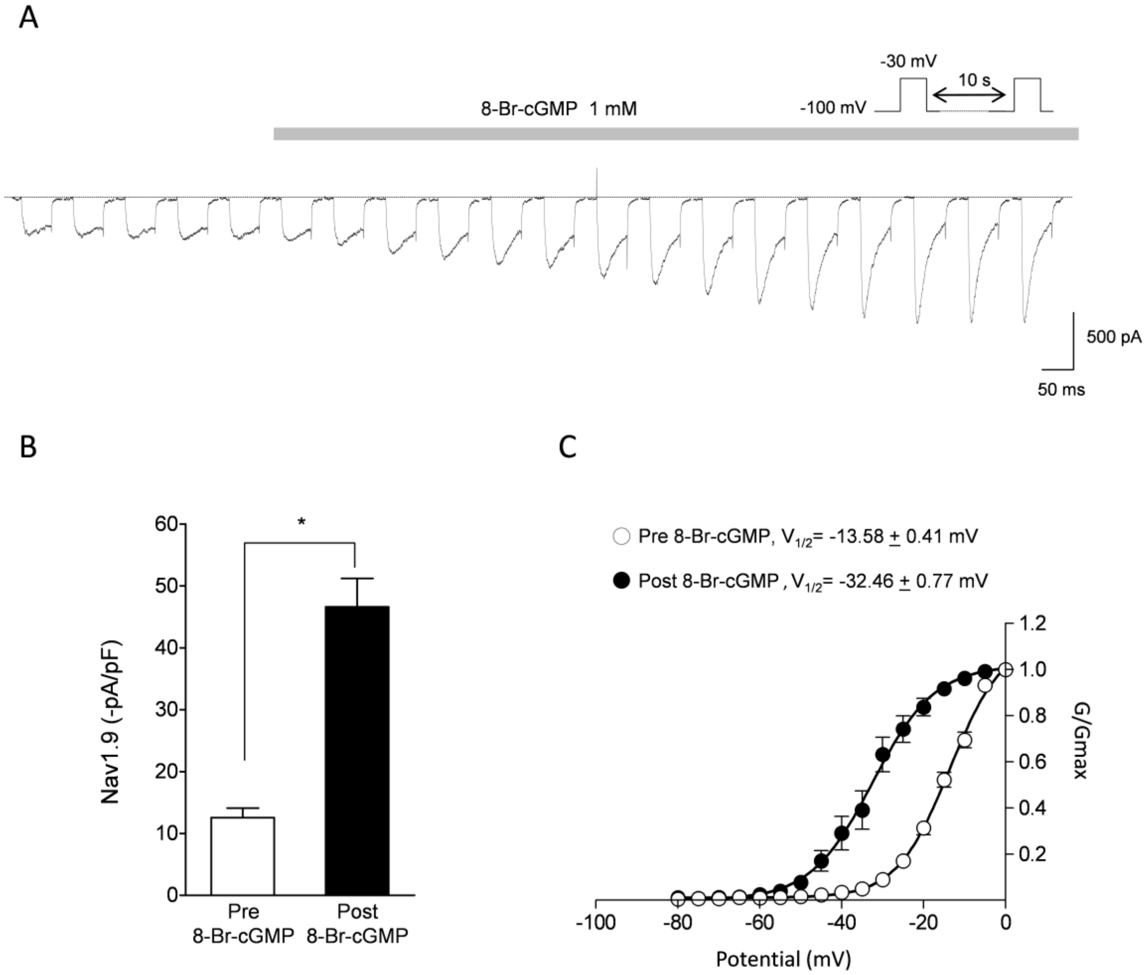

Supplementary Figure 6. The cell-permeable cGMP analog 8-Br-cGMP activates Nav1.9 in dural afferent neurons

(A) Effect of the superfusion of 8-Br-cGMP (1 mM) on Nav1.9 current recorded in a dural afferent neuron from a saline-treated Nav1.8$^{-/-}$ mouse. Currents were evoked from a holding potential of -100 to -30 mV. CsCl-only-based patch pipette solution. TGs were cultured at day 21.

(B) Histogram showing the change in mean Nav1.9 current density before and after application of 8-Br-cGMP (1 mM) in dural afferent neurons (n=12) from saline-treated Nav1.8$^{-/-}$ mice. TGs were cultured at day 21. *p<0.05 with Wilcoxon matched paired test.

(C) Averaged activation curves for Nav1.9 determined before and after 8-Br-cGMP application in dural afferent neurons (n=12). 8-Br-cGMP caused a negative shift of ~19 mV in the activation $V_{1/2}$ value for Nav1.9.

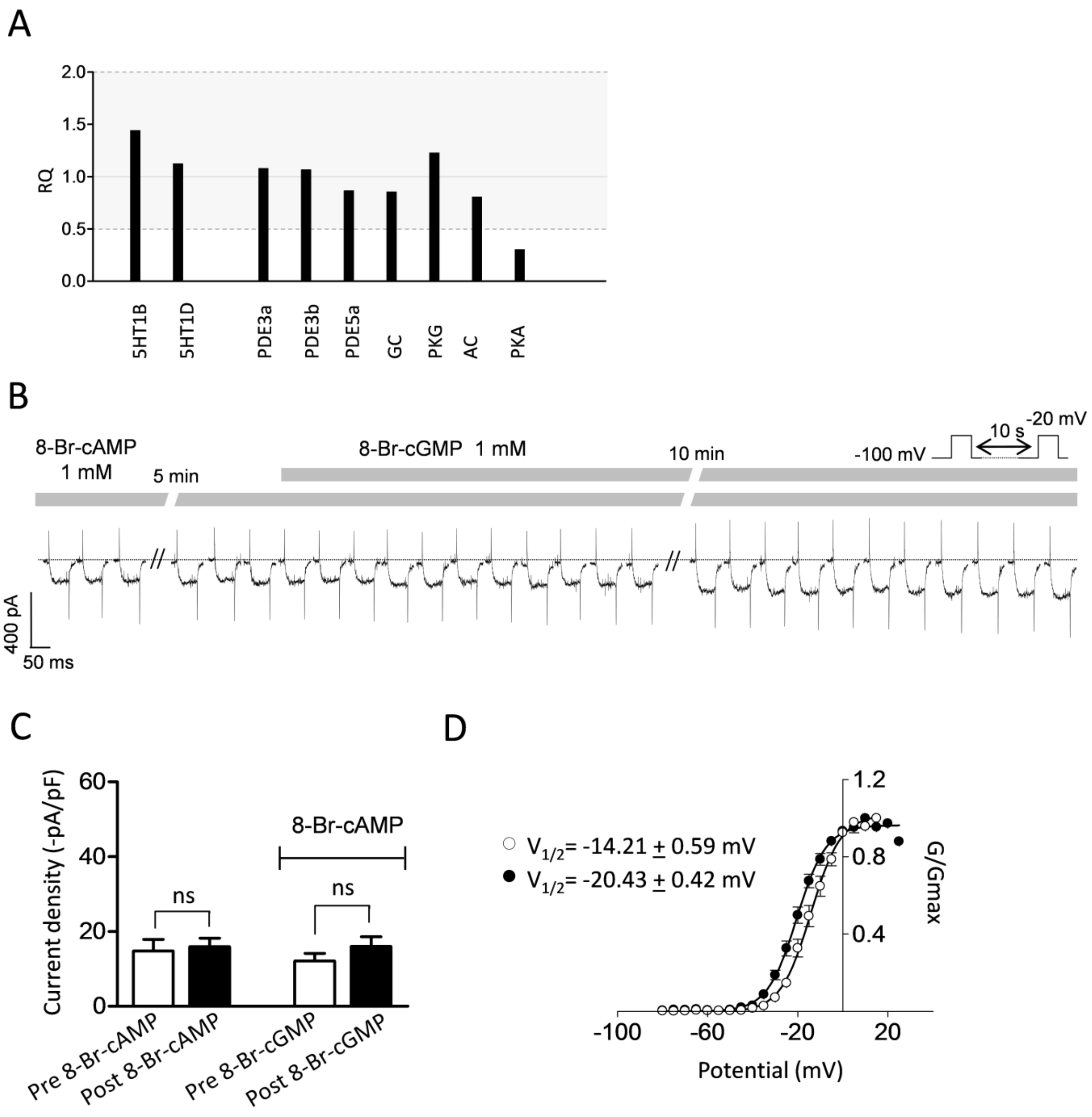

Supplementary Figure 7. PKA downregulation promotes NO coupling to Nav1.9 channels

(A) Transcriptional expression of the receptors 5HT1B and 5HT1D, the cyclic nucleotide phosphodiesterases PDE3a, PDE3b, PDE5a, the sGC, AC-III and the kinases PKG-I and PKA-Cα. The level of the gene of interest was measured in sumatriptan-treated TG neurons relative to the level of gene expression in control (saline) TG neurons (2-ΔΔCt method). TGs were dissected out at day 21 in mice treated with saline solution or sumatriptan. GAPDH was used as house keeping gene.

(B) Effect of pretreatment of 8-Br-cAMP (1 mM) on 8-Br-cGMP-induced activation of Nav1.9 current recorded in a dural afferent neuron from a saline-treated Nav1.8$^{-/-}$ mouse. Currents were evoked from a holding potential of -100 to -20 mV. CsCl-only-based patch pipette solution. TGs were cultured at day 21.

(C) Mean Nav1.9 current density before and after application of 8-Br-cAMP (1 mM) in dural afferent neurons (n=12, saline-treated Nav1.8$^{-/-}$ mice) and before and after application of 8-Br-cGMP (1 mM) in dural afferent neurons (n=12) pre-treated with 8-Br-cAMP (1 mM) for 10 min. TGs were cultured at day 21. *p<0.05 with Wilcoxon matched paired test.

(D) Averaged activation curves for Nav1.9 determined in the presence of 8-Br-cAMP before (open symbols) and after (filled symbols) 8-Br-cGMP application in dural afferent neurons (n=12, Nav1.8$^{-/-}$ mice).

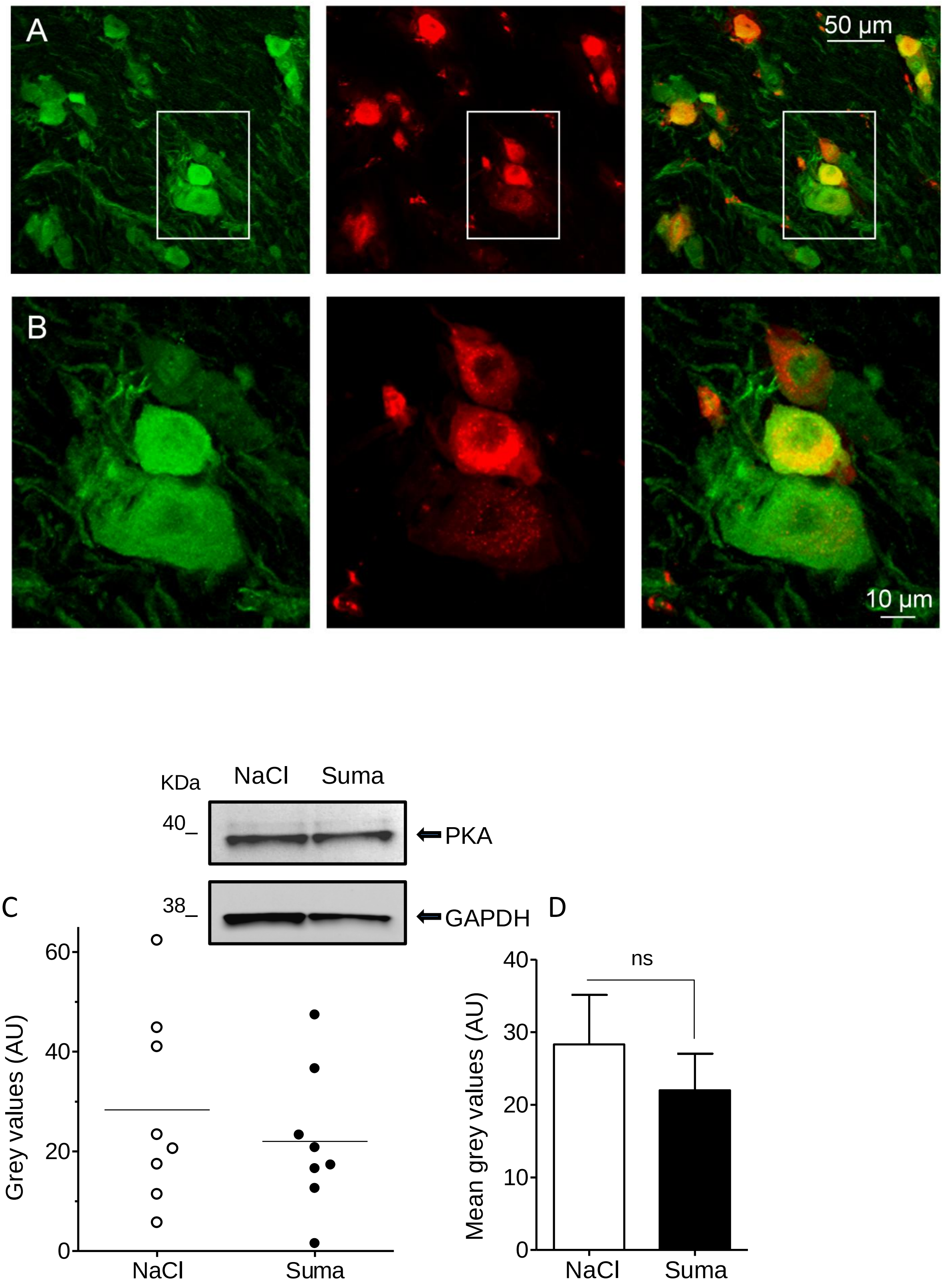

Supplementary Figure 8. Dural afferent neurons show high levels of activated PKA

(A-B) DiI-retrogradely labeled TG neurons (red) were stained with T197, an antibody that recognizes the phosphorylated active form of PKA (green). TGs were collected two days after DiI application through a cranial window in the parietal bone. Note, that some DiI-positive, small diameter, TG neurons showed a high level of constitutively activated PKA. Images are projections of 8 consecutive optical sections spanning 9 µm. Rightmost panel: merged images.

(C-D) Individual (C) or mean (D) Western blot densitometry values of blots probed with anti-T197 PKA from sumatriptan- and saline-treated TGs collected at day 21. Single dot represents 2 TGs from a single mouse. Loading, 20 µg/lane. ns, not significant (p = 0. 46, Mann-Whitney test). Inset shows a Western blot probed with anti-T197 PKA and anti-GAPDH (as control). Note that normalization of PKA signal to GAPDH signal shows a 15 ± 2% decrease in T197 PKA expression in SUMA-treated mice (n=4) compared to saline-treated mice (n=4), although this did not reach statistically significant level (Mann-Whitney test).

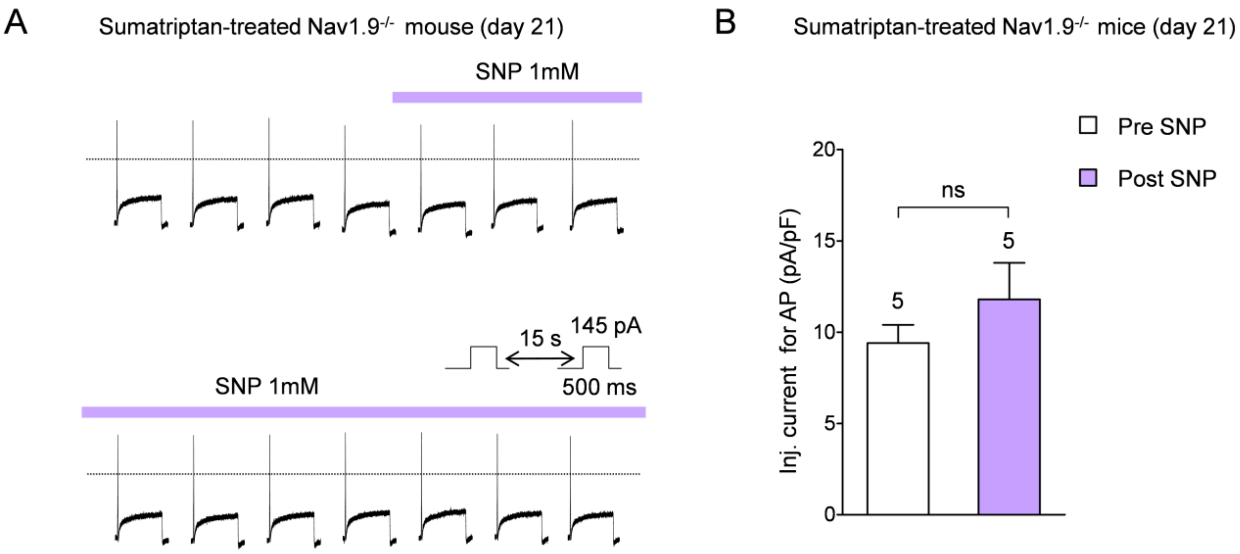

Supplementary Figure 9. SNP has no excitatory effects in dural afferent neurons from sumatriptan-treated Nav1.9$^{-/-}$ mice

(A) Current-clamp responses of DiI^{+} dural afferent neurons from Nav1.9$^{-/-}$ mice treated with sumatriptan. Voltage responses were evoked by 500 ms-depolarizing pulses. The horizontal bars indicate the time and duration of SNP application (1 mM). KCl-based intracellular solution. TGs were cultured at day 21.

(B) Current threshold for AP (normalized to the cell membrane capacitance) before and after SNP exposure in sumatriptan-treated Nav1.9$^{-/-}$ dural afferent neurons. ns, not significant with Wilcoxon matched paired test.

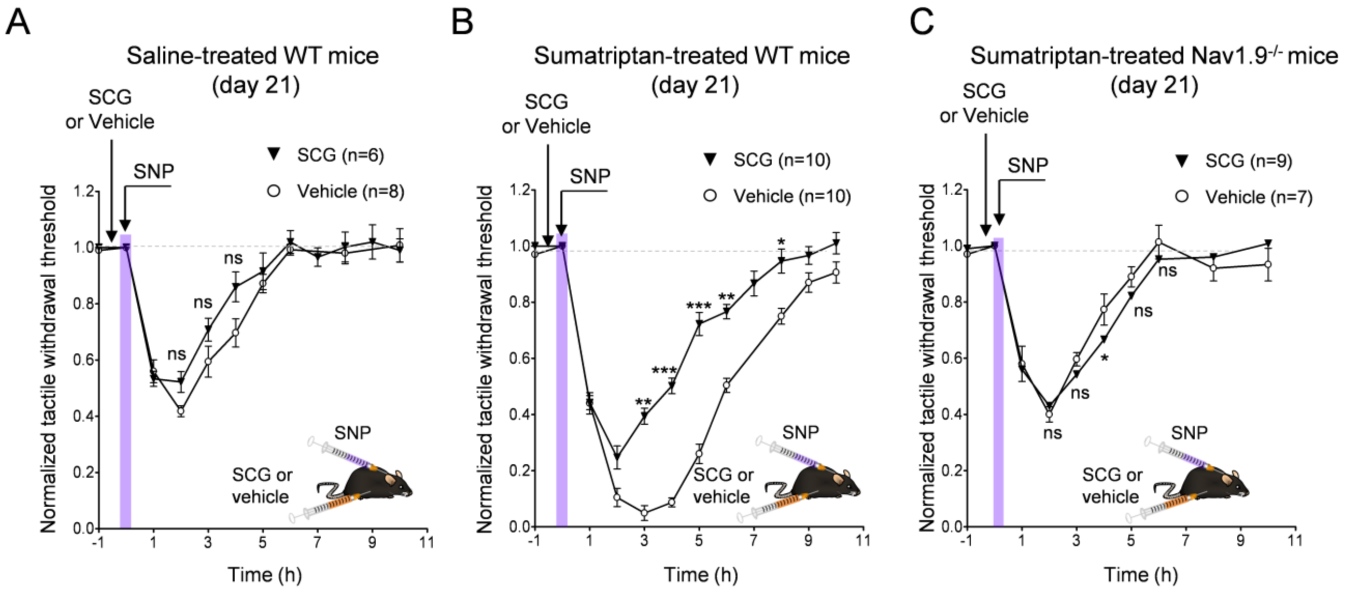

Supplementary Figure 10. Inhibition of mast cell degranulation reduces SNP-induced heightened allodynia

(A-C) Effect of intraperitoneal injection of the MC stabilizing agent SCG (10 mg/kg) on the SNP-induced allodynia in saline-treated WT mice (A), sumatriptan-treated WT mice (B), and sumatriptan-treated Nav1.9$^{-/-}$ mice (C). Injection of SCG or the vehicle (NaCl) was achieved 30 min prior to subcutaneous injection of SNP (0.03 mg/kg). Experiments were performed at day 21. ns, not significant, *p<0.05, **p<0.01, ***p<0.001 compared to vehicle-injected mice with Mann-Whitney non-parametric test.

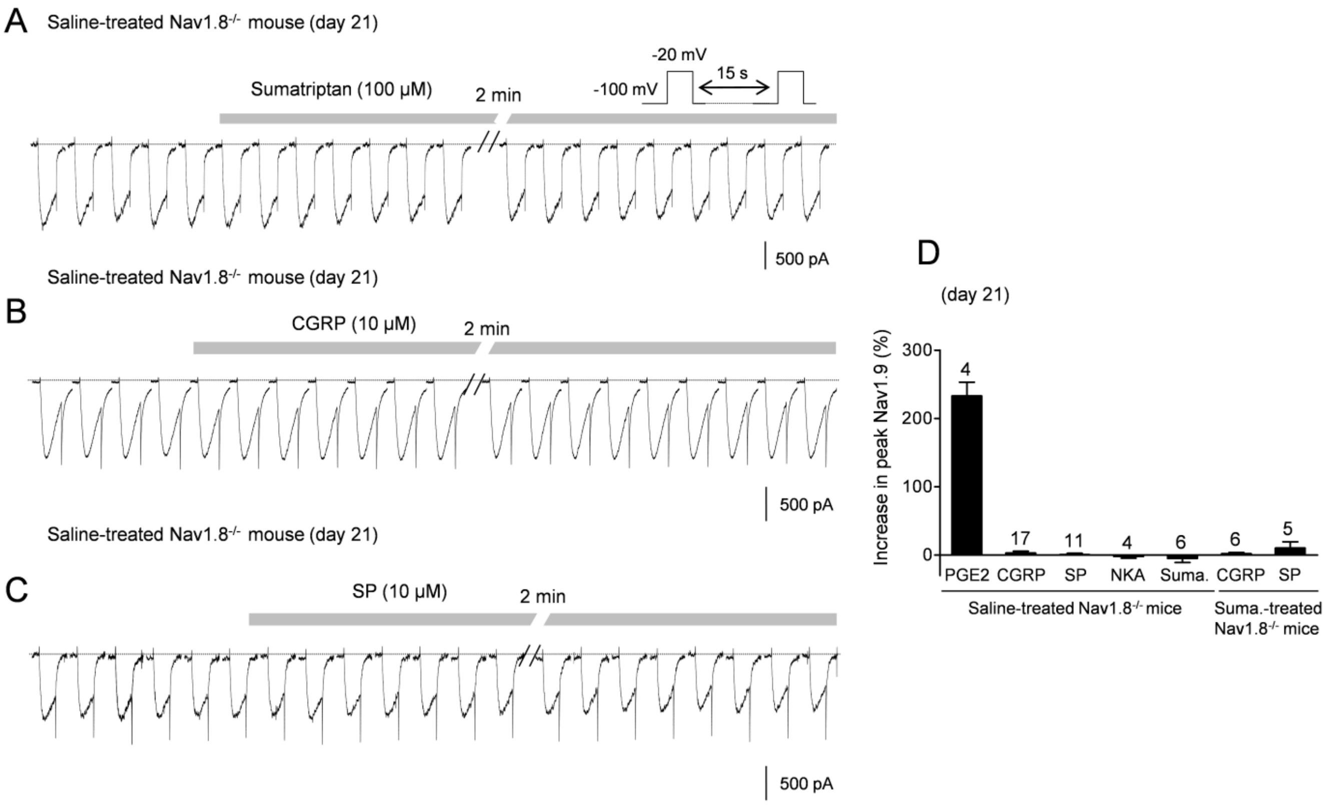

Supplementary Figure 11. CGRP and substance P have no effects on Nav1.9 in dural afferent neurons from saline and sumatriptan-treated mice

(A-C) Lack of effect of sumatriptan (100 µM, A), CGRP (10 µM, B) and SP (10 µM, C) on Nav1.9 current in dural afferent neurons from saline-treated Nav1.8$^{-/-}$ mice. Currents were evoked from a holding potential of -100 to -20 mV every 15 s. CsCl-only-based patch pipette solution. Cell culture made at day 21.

(D) Histogram showing the mean increase in Nav1.9 peak current induced by PGE2 (500 nM, as positive control), CGRP (10 µM), SP (10 µM), neurokinin-A (Neuro-A, 10 µM) and sumatriptan (Suma, 100 µM) in dural afferent neurons from either saline or sumatriptan-treated Nav1.8$^{-/-}$ mice. Cell culture made at day 21.
