# Supplementary material for: Maladaptive activation of Nav1.9 channels by nitric oxide causes triptan-induced medication overuse headache
Source: Nat Commun. 2019 Sep 18;10:4253. doi: 10.1038/s41467-019-12197-3 (PMC6751217; doi:10.1038/s41467-019-12197-3)
Supplement: Supplementary file 3 — Reporting Summary [file 41467_2019_12197_MOESM3_ESM.pdf]

nature research

Corresponding author(s): NCOMMS-18-36489A
Last updated by author(s): Aug 15, 2019

Reporting Summary

Nature Research wishes to improve the reproducibility of the work that we publish. This form provides structure for consistency and transparency in reporting. For further information on Nature Research policies, see Authors & Referees and the Editorial Policy Checklist .

Statistics

For all statistical analyses, confirm that the following items are present in the figure legend, table legend, main text, or Methods section.

n/a	Confirmed	
☐	☒	The exact sample size (n) for each experimental group/condition, given as a discrete number and unit of measurement
☐	☒	A statement on whether measurements were taken from distinct samples or whether the same sample was measured repeatedly
☐	☒	The statistical test(s) used AND whether they are one- or two-sided<br>*Only common tests should be described solely by name; describe more complex techniques in the Methods section.*
☒	☐	A description of all covariates tested
☐	☒	A description of any assumptions or corrections, such as tests of normality and adjustment for multiple comparisons
☐	☒	A full description of the statistical parameters including central tendency (e.g. means) or other basic estimates (e.g. regression coefficient) AND variation (e.g. standard deviation) or associated estimates of uncertainty (e.g. confidence intervals)
☐	☒	For null hypothesis testing, the test statistic (e.g. F, t, r) with confidence intervals, effect sizes, degrees of freedom and P value noted<br>*Give P values as exact values whenever suitable.*
☒	☐	For Bayesian analysis, information on the choice of priors and Markov chain Monte Carlo settings
☒	☐	For hierarchical and complex designs, identification of the appropriate level for tests and full reporting of outcomes
☐	☒	Estimates of effect sizes (e.g. Cohen's d, Pearson's r), indicating how they were calculated

Our web collection on statistics for biologists contains articles on many of the points above.

Software and code

Policy information about availability of computer code

Data collection	PCLAMP 9.2 (Axon Instruments Inc.)
Data analysis	Clampfit 9.2 (Molecular Devices), PRISM 4.0 (GraphPad), Origin 7.0 (OriginLab), Adobe Photoshop (Adobe Systems, San Jose, CA), Packwin software (Panlab, Inc. Harvard apparatus).

For manuscripts utilizing custom algorithms or software that are central to the research but not yet described in published literature, software must be made available to editors/reviewers. We strongly encourage code deposition in a community repository (e.g. GitHub). See the Nature Research guidelines for submitting code & software for further information.

Data

Policy information about availability of data

All manuscripts must include a data availability statement. This statement should provide the following information, where applicable:

- Accession codes, unique identifiers, or web links for publicly available datasets
- A list of figures that have associated raw data
- A description of any restrictions on data availability

The authors declare that data supporting the findings of this study are available within the paper and the supplementary information file.

Field-specific reporting

Please select the one below that is the best fit for your research. If you are not sure, read the appropriate sections before making your selection.

☒ Life sciences ☐ Behavioural & social sciences ☐ Ecological, evolutionary & environmental sciences

Life sciences study design

All studies must disclose on these points even when the disclosure is negative.

Sample size	We did not systematically performed calculation to justify sample size but our sample sizes are similar to those reported in previous publications. However, in some behavioral experiments we performed sample size calculations using the software G*Power (http://www.gpower.hhu.de/). We found that sample sizes with a minimum of 8-13 animals were necessary in most experiments, hence our typical sample sizes of 10-15 animals in most tests. For electrophysiological experiments, more than 10 cells were typically analyzed, except for some technically challenging experiments.
Data exclusions	No analyzed data were excluded. However, some (3) animals were discarded from the study because they developped a local inflammation following minipump implantation.
Replication	To verify reproducibility of the experimental findings, each tested condition was assessed in different experiments, elapsed by several weeks or months and always associated with its own control performed contemporaneously.
Randomization	Randomization was used in behavioral experiments.
Blinding	The behavioral experiments in which animals were treated with sumatriptan or saline solution chronically (with minipumps), and injected at day 21 with SNP or vehicle were made blind; i.e. the investigator was not aware of the content of the minipump and of the nature of the solution (SNP or vehicle) injected on day 21.

Reporting for specific materials, systems and methods

We require information from authors about some types of materials, experimental systems and methods used in many studies. Here, indicate whether each material, system or method listed is relevant to your study. If you are not sure if a list item applies to your research, read the appropriate section before selecting a response.

Materials & experimental systems

n/a	Involved in the study	
☐	☒	Antibodies
☒	☐	Eukaryotic cell lines
☒	☐	Palaeontology
☐	☒	Animals and other organisms
☒	☐	Human research participants
☒	☐	Clinical data

Methods

n/a	Involved in the study	
☒	☐	ChIP-seq
☒	☐	Flow cytometry
☒	☐	MRI-based neuroimaging

Antibodies

Antibodies used	Anti-peripherin 1/400 (mouse monoclonal, Millipore, Temecula, CA); anti-NF200 1/600 (chicken polyclonal, Aves Labs, Tigard, OR); anti-CD31 (1/400, rat polyclonal, BD Biosciences, Belgium); anti-Nav1.9 L23, (1/100, rabbit polyclonal) (Padilla et al., 2007), anti-CGRP (1/300, goat polyclonal, AbCam), anti-PKA antibody (rabbit polyclonal #ab75991, AbCam).<br>Secondary antibodies were: Alexa Fluor 674-conjugated donkey anti-mouse (1/400, Life Technologies), TRITC-conjugated donkey anti-rabbit (1/400, Jackson ImmunoResearch, Suffolk, UK), TRITC-conjugated donkey anti-rat (1/100, Jackson ImmunoResearch), Alexa Fluor 488-conjugated donkey anti-goat (1/200, Life Technologies).
Validation	Our anti-Nav1.9 antibody has been validated using Nav1.9-KO tissues and previously published in Padilla et al., (Expression and localization of the Nav1.9 sodium channel in enteric neurons and in trigeminal sensory endings: implication for intestinal reflex function and orofacial pain. Padilla F, Couble ML, Coste B, Maingret F, Clerc N, Crest M, Ritter AM, Magloire H, Delmas P<br>Mol Cell Neurosci. 2007 May; 35(1):138-52.)

Animals and other organisms

Policy information about studies involving animals; ARRIVE guidelines recommended for reporting animal research

Laboratory animals	Mice (10-12 week adult males, C57Bl/6J background) used were Nav1.9-/-, Nav1.8-/- and their wild-type (WT) littermates.
Wild animals	The study did not involve wild animals
Field-collected samples	The study did not involve samples collected from the wild
Ethics oversight	This project was approved by the institutional review board of the regional ethic committee (Comité Régional d'Ethique en Matière d'Expérimentation Animale). All animals were used in accordance with the European Community guiding in the care and

use of animals (2010/63/UE).

Note that full information on the approval of the study protocol must also be provided in the manuscript.
