# Supplementary material for: Maladaptive activation of Nav1.9 channels by nitric oxide causes triptan-induced medication overuse headache
Source: Nat Commun. 2019 Sep 18;10:4253. doi: 10.1038/s41467-019-12197-3 (PMC6751217; doi:10.1038/s41467-019-12197-3)
Supplement: Supplementary file 2 — Peer Review [file 41467_2019_12197_MOESM2_ESM.pdf]

Reviewers' comments:

Reviewer #1 (Remarks to the Author):

A technically excellent study of the role of Nav1.9 in NO-induced dural nociceptor sensitization in a model of medication overuse headache (MOH) employing well-integrated genetic, biochemical, electrophysiological and behavioral studies. Theur use of triptan-induced MOH makes these studies highly relevant to our understanding of the response of migraine to one of its primary treatment modalities. As our current knowledge of the mechanism of MOH is extremely limited, their results provide an important mechanistic model for the treatment of this common and debilitating pain syndrome. My suggestions mainly relate to ways in which their studies might be better integrated into the relevant clinical literature.

The title probably should indicate that they are studying a model of MOH.

The relative contribution of serotonin and histamine in the rodent mast cell, as triptans act at serotonin receptors.

Use of male mice in a model of a female predominant pain syndrome.

Clinical observation that "MOH does not develop in persons without a history of headache when medication is being used for other conditions, such as inflammatory diseases," as it relates to the present studies.

PGE2 ◊ cAMP ◊ PKA is a class pathway to sensitize nociceptors/

Congenital insensitivity to pain can be induced by loss of NaV1.9 function. Thus, presumably baseline thresholds are elevated in their knockout mice (hence their use of normalized threshold). This should be noted explicitly.

The trigger of headache by NO donors, in patients with migraine has a delayed onset, on the order of hours. I don't know if this is true for MOH.

Sodium cromoglycate has not been useful for the treatment of migraine.

The spectrum of the light source?

Page 4. (TTX) Na+ currents ◊ (TTX) sensitive Na+ currents

An outstanding contribution that substantially advances our understanding of medicine induced headache.

Reviewer #2 (Remarks to the Author):

The authors are investigating the role of Nav1.9 in medication over-use headache which is an important clinical problem. They develop a model of MOH based on chronic dosing with sumatriptan and suggest a mechanism whereby this results in reduced expression of PKAC-α, this is associated with enhanced coupling between NO and Nav1.9, trigeminal neuron hyper-excitability and behavioural hypersensitivity to mechanical stimuli, photo and phonophobia. The model is definitely interesting, the identification of Nav1.9 as a key hub in MOH would be a significant advance for the field. There are however a number of important weaknesses that need to be addressed:

Major points:
1. Gender: All the mice used in the study were male yet as the authors point out migraine/MOH is much commoner in females. Why were female mice not studied? This is a significant deficiency in the study and at least the major finding that Nav1.9 has a key role in MOH (at a behavioral level) should be replicated female mice.

2. Behavioral analysis and blinding: In the behavioral testing (eg. Fig 2). Why is a normalized mechanical withdrawal threshold given, what is it normalized to? The authors refer to a previous publication but this gives absolute values not normalized data. It is preferable in my view to give absolute values or at least a more detailed and transparent explanation (eg. is this because Nav1.9 -/- have different baseline values?). Peri-orbital testing data is only shown at day 21 given that this is the most relevant aspect for MOH/migraine it should also be given at earlier timepoints (as it has been for hindpaw).

Re. blinding in the reporting section the statement is that 'The behavioral experiments in which animals were treated with sumatriptan or saline solution chronically (with minipumps), and injected at day 21 with SNP or vehicle were made double blind; i.e. the investigator was not aware of the content of the minipump, nor was he aware of the solution (SNP or vehicle) he was injecting on day 21.' I was confused by this as All behavioral experiments should be performed blind. Was the investigator also blind to genotype? A statement regarding blinding needs to be made in the manuscript (please note that it doesn't make sense referring to a double blind experiment in animals).

3. Mechanism by which chronic sumatriptan enhances coupling between NO and Nav1.9: Is there direct evidence that chronic sumatriptan treatment enhances NO release and there are also assays available for assessing cGMP? There is a suggestion that the sumatriptan leads to reduced PKAC-α and this results in less inhibition of cGMP signalling. It would be helpful to look at this further at protein level for instance with western blot analysis to back up the q-PCR data and also looking at the activated phospho T197 PKA form which is currently only examined with immunostaining.

4. The level of statistical reporting could be improved throughout: See comments on blinding above. In the methods section some general comments on the statistical tests used are provided. However it is essential that the exact test used for that specific experiment is given in each case (ie in the actual results we don't know when a t-test, versus one way versus two way ANOVA was used etc). In 'A description of any assumptions or corrections, such as tests of normality and adjustment for multiple comparisons'. NA is given. The data should always be tested for normality and I presume that this was done? This should be stated in the statistics section. 'Sample size was typically determined/chosen to make statistical analysis relevant. For electrophysiological experiments, more than 10 cells were typically analyzed, except for some rare technically challenging experiments (n=6). The rationale for why these sample sizes are sufficient is because it is compatible for the chosen statistical tests. Behavioral studies used a minimum of 10 animals in most tests, to minimize variability between animals. As a rule, no conclusions were derived from small n numbers.' This is not a great justification of sample size. We don't choose sample size just to suit the statistical test undertaken. The sample size chosen relates to the variance in the data, the effect size that we think is meaningful as well as the significance level (alpha). According to the ARRIVE guidelines there should be a power calculation performed at the outset to justify sample size. I suggest that the authors rethink this section and perform some proper sample size calculations albeit post hoc.

Minor points
1. Supplemental figure 3 it looks to me that the hypersensitivity induced by sumatriptan is less in the Nav1.8-/- versus WT mice (panel A). What is the result of direct statistical comparison of these two groups?

2. I may have missed it but more information needs to be given re mice expressing β-Gal at the

SCN11a locus. At least a reference of where/how this line was generated should be given (this applies to all the transgenic lines). The authors state that double staining of CGRP and Nav1.9 could not be undertaken. Good antibodies to β Galactosidase are available meaning that it would be simple to assess both the co-expression of Nav1.9 immunostaining with β Gal and also β Gal and CGRP.
3. In results page 7. 'Neither the mean Nav1.9 peak current amplitude (Fig 4B), nor the level of Nav1.9 mRNA…' Do they mean to say 'Neither the mean Nav1.9 peak current density?' which is the top graph in the panel?
4. In current clamp analysis of dural afferents in Figure 5. Do any of these neurons develop spontaneous activity in response to SNP? Is there a change in action potential waveform in response to SNP?

Reviewer #3 (Remarks to the Author):

Bonnet et al. report the role of the voltage-gated sodium channel Nav1.9 in medication overuse headache (MOH). Nav1.9 is a channel involved in pain sensation and generates a persistent sodium current to modulate neuronal activity. It´s mutations are associated with different human pain disorders. In mice, the authors show here that chronic use of triptans induces MOH through abnormal activation of meningeal Nav1.9 channels. Bonnet and colleagues administer the 5-HT receptor agonist sumatriptan, which is approved for acute therapy in migraine and in cluster headache, and show that animals chronically treated with the drug display increased responsiveness / abnormal activation of Nav1.9 to nitric oxide (NO). NO is a well-known trigger for headache and migraine. Phenotypic readout for the "migraine-like symptoms" in mice are generalized allodynia, photophobia, and phonophobia. The authors suggest that Nav1.9-mediated release of neuropeptides from meningeal nociceptors involves degranulation of mast cells and causes inflammatory pain.

This is a very interesting paper dealing with a highly relevant topic and has implications for translational approaches in human pain therapy. The findings are new. Methods and experimental approaches are state-of-the-art and I have only few minor suggestions:

Migraine occurs at a 3:1 female to male ratio. In their experiments, the authors report the results from male mice. Can the authors maybe comment whether sex-specific differences have also been observed using female mice? This would be a very interesting point to address, however, repeating all experiments with a cohort of female mice would exceed the aim of the present study.

Can the authors speculate whether the application of currently available sodium-channel blockers in combination with sumatriptan or other headache medications would be beneficial?

Methods: Please provide more details on the generation of the Nav1.8 and Nav1.9 KO-mice used in the study.

NCOMMS-18-36489

Point-by-point reply to Reviewers' comments.

Reviewer #1 (Remarks to the Author):

A technically excellent study of the role of Nav1.9 in NO-induced dural nociceptor sensitization in a model of medication overuse headache (MOH) employing well-integrated genetic, biochemical, electrophysiological and behavioral studies. Theur use of triptan-induced MOH makes these studies highly relevant to our understanding of the response of migraine to one of its primary treatment modalities. As our current knowledge of the mechanism of MOH is extremely limited, their results provide an important mechanistic model for the treatment of this common and debilitating pain syndrome. My suggestions mainly relate to ways in which their studies might be better integrated into the relevant clinical literature.

We thank the referee for his/her very helpful comments.

The title probably should indicate that they are studying a model of MOH.

We refer now to Triptan in the title, which reads "Maladaptive activation of Nav1.9 channels by Nitric Oxide causes Triptan-induced Medication Overuse Headache".

The relative contribution of serotonin and histamine in the rodent mast cell, as triptans act at serotonin receptors.

This is a good point. 5-HT is implicated in enhancing inflammatory reactions of skin, lung and many other tissues. Mast cells (MCs) are known to express serotonin receptors. However, in mouse bone marrow-derived MCs and human CD34-derived MCs there is no evidence that 5-HT degranulates MCs or modulates IgE-dependent activation but instead 5-HT seems to promote MC adherence to fibronectin and MC migration. 5-HT_{1A} is the principal receptor mediating the effects of 5-HT in these MCs (Kushnir-Sukhov et al., *J Immunol* 2006; 177:6422-6432). Thus, these data suggest that 5-HT may sustain inflammation by increasing MCs at the site of tissue injury, but not necessarily through degranulation and release of histamine.

Another study dealing with MCs from the guinea pig small intestine and segments of human jejunum also suggested that 5HT effects are mediated via the 5-HT1A receptor. At variance, this study also showed that stimulation of 5-HT1A can degranulate MCs and release histamine (Wand et al., *Am J Physiol Gastrointest Liver Physiol* 304: G855–G863, 2013). It is also well known that treatment with anti-5-HT1AR diminishes the severity of contact allergy in experimental animals, an effect mediated by mast cells; while an agonist of this same receptor reduces the stress level and relieves pruritus in patients with atopic dermatitis, a disease also involving mast cells.

Sumatriptan is a 5-HT_{1D}/5-HT_{1B} receptor agonist (Razzaque Z, Heald MA, Pickard JD, et al., 1999, Br J Clin Pharmacol. 47: 75–82) that is supposed not to be active on the 5-HT_{1A}

receptor. Therefore, although one cannot definitely rule out that sumatriptan interacts with MCs, the current knowledge supports the idea that 5HT action on MCs is mediated by the 5-HT1A receptor.

A sentence has been added in the discussion on page 14. It reads "*Because MCs express a variety of 5-HT receptors, it is possible that chronic treatment with sumatriptan reinforces the possibility that MCs respond to peptide-induced degranulation. However, the reported effects of 5-HT on MCs have generally been found to be mediated by 5-HT1A and not by 5HT1B/D receptors (47).*"

Use of male mice in a model of a female predominant pain syndrome.

This is an important point, indeed, which has been raised by the 3 referees and the Editor. It is something we had in mind because the male: female ratio is about 1: 3.

We have now completed a new series of experiments in which the effects of chronic infusion of sumatriptan (or NaCl) and injection of SNP (or NaCl) at day 21 were tested on WT (n=20) and Nav1.9 KO (n=19) mouse females. As before, this series of experiments was made blind.

We treated chronically mouse females with sumatriptan (0.6 mg/kg/day, as with males) and assessed quantitatively generalized mechanical allodynia using von Frey filaments. We found that sumatriptan infusion but not saline solution (vehicle, 0.9%), decreased withdrawal thresholds to tactile stimuli applied to the hind paws of WT female mice. Females appeared more sensitive than males as hind paw withdrawal threshold was sensibly lower and sensory threshold was slower to recover. Similar to males, sumatriptan-treated WT females showed enhanced mechanical hypersensitivity at day 21 in response to SNP injection (0.03 mg/kg). This heightened allodynia was fully prevented in Nav1.9 KO female mice.

These data are now included in the result section on page 6 and in supplementary Figure 2B-D; it reads "*Because females have increased risk of developing migraine and MOH, we tested whether infusion of sumatriptan for 6 days (0.6 mg/kg/day) produced mechanical hypersensitivity and latent sensitization to NO in WT female mice as observed for the opposite gender. Chronic sumatriptan produced a strong reduction in mechanical withdrawal thresholds of the hind paw relative to saline-treated WT female mice and to sumatriptan-treated Nav1.9$^{-/-}$ female mice (Supplemental Fig. 2B). Injection of SNP (0.03 mg/kg) at day 21 once sensory thresholds had returned to pre-sumatriptan baseline values caused significantly stronger mechanical allodynia in sumatriptan-treated WT female mice compared to saline-treated WT female mice (Supplemental Fig. 2C). SNP-induced heightened mechanical allodynia was absent in sumatriptan-treated Nav1.9$^{-/-}$ female mice (Supplemental Fig. 2D), reaching similar amplitude to that caused by SNP in saline-treated Nav1.9$^{-/-}$ female mice (Supplemental Fig. 2D). This series of experiments shows that Nav1.9, as observed in male mice, had no role in SNP-induced allodynia in saline-treated animals, but contributes to the heightened SNP allodynia in sumatriptan-treated female mice*."

Clinical observation that "MOH does not develop in persons without a history of headache when medication is being used for other conditions, such as inflammatory diseases," as it relates to the present studies.

This notion has been proposed by specialists in the field (Espen Saxhaug Kristoffersen, Christofer Lundqvist). However, we could not find a report in which people with other conditions (arthritis or inflammatory diseases) were asked to take medication such as simple analgesics more than 15 days per month or a combination analgesics more than 10 days per month for 3 months or more (criteria for MOH, cf Headache Classification Committee of the International Headache Society, 2013), and were then evaluated for headache. Having said that, it is true that if pre-existent headache disorder is required to develop MOH, then treating, presumably non-migrainous, mice with sumatriptan may not be a 'perfect' model of MOH. This is clearly the limitation of our mouse model, and of most models dealing with migraine/headache, perhaps with the exception of genetic models. However, because a clear connection has been made between headache-specific pain pathways and headache medication effects in generating a more chronic pain, our model at least addresses the mechanisms of chronic headache medication effects, which resemble phenomena seen in dependence/addiction processes.

PGE2 – cAMP – PKA is a class pathway to sensitize nociceptors

cAMP/PKA pathway is indeed known to sensitive and modulates the pain pathway. It is well established in DRG neurons but the situation is not that clear in TG neurons. Levy and Strassman (2002; *Journal of Physiology* (2002), 538.2, *pp.* 483–493) have shown that the cAMP-PKA cascade is involved in sensitization of dural mechanonociceptors through different mechanisms operating in separate neuronal populations. But they also showed that 34% of meningeal mechanosensitive units were not sensitized by local application to the dura of dibutyryl adenosine 3,5,-cyclic monophosphate (dbcAMP), a stable membrane-permeant cAMP analogue. Activation of PKA- and B-Raf-dependent p38 MAPK pathways in mouse TG neurons has also been shown to decrease membrane excitability through the stimulation of A-type K^+ channel (Zhao et al., Cell Signal. 2016 Aug;28(8):979-88). Cannabinoid agonists are known to inhibit TRPV1 in trigeminal ganglion neurons though PKA and PKC pathways (Wang et al., Neurol Sci. 2012 Feb;33(1):79-85). So, PKA pathways (in the cell body? in the terminals?) may also have inhibitory effects.

Our data shows that cAMP inhibits the coupling between NO and Nav1.9, so the resulting effect is a decrease in excitability driven by Nav1.9, but this does not exclude other actions of cAMP that may promote excitation. We found that PGE2 has excitatory effects on dural nociceptors through its potentiation of Nav1.9, but we have evidence that PKA does not mediate its effects.

Congenital insensitivity to pain can be induced by loss of NaV1.9 function. Thus, presumably baseline thresholds are elevated in their knockout mice (hence their use of normalized threshold). This should be noted explicitly.

Insensitivity to pain linked to Nav1.9 mutation (L811P, L1302F) in humans is probably due gain-of-function but not loss-of-function properties of the channel (Huang et al., J Clin Invest.

2017 Jun 30; 127(7): 2805–2814). Nav1.9 mutations that evoke small degrees of membrane depolarization cause hyperexcitability and familial episodic pain disorder or painful neuropathy, while Nav1.9 mutations evoking larger membrane depolarizations generate hypoexcitability (by inactivating the spike-generating system) and insensitivity to pain.

In mice, it is well established by different groups (S. Waxman, J.N. Wood; BT Priest, etc..) that Nav1.9 KO mice have quite normal sensory (noxious heat & mechanical stimuli) thresholds. The only acute phenotype of Nav1.9 KO mice is on noxious cold (Lolignier et al., the Nav1.9 channel is a key determinant of cold pain sensation and cold allodynia, Cell Rep. 2015 11(7):1067-78.). Actually, Nav1.9 channels have a low constitutive activity under normal conditions but are particularly active upon inflammation.

The first reason of using normalized threshold is because of within-strain variation of mechanical withdrawal threshold (from 0.4 to >1 g) even in standardized mouse environment. This also helps comparison between genotypes.

The trigger of headache by NO donors, in patients with migraine has a delayed onset, on the order of hours. I don't know if this is true for MOH.

We think so, but could not find a specific study in the literature. NO can cause immediate headache in most normal people and cause a delayed headache (5-6 hr after exposure) in people with 1) migraine without aura, 2) chronic tension-type headache, infrequent episodic tension-type headache and frequent episodic tension-type headache. People with cluster headache develop a cluster headache attack 1-2 hours after intake.

Sodium cromoglycate has not been useful for the treatment of migraine.

Sodium cromoglycate has been shown to be effective in controlling gastrointestinal symptoms, but is less effective in other body systems. It is possibly not reaching appropriate concentrations in the meninges. It is poorly absorbed through all body surfaces apart from the bronchial mucosa. When administered orally between 0.8% and 1% is absorbed systemically. However, inhaled sodium cromoglycate has been shown to decrease the symptoms of bone pain, fatigue and headache (Edwards AM, Hagberg H. Oral and inhaled sodium cromoglicate in the management of systemic mastocytosis: a case report. J Med Case Rep. 2010;4:193. Published 2010 Jun 26. doi:10.1186/1752-1947-4-193) and in some cases has also been reported to exert a protective effect on hypersensitivity mechanisms as well as the symptoms of migraine (Monro J, Carini C, Brostoff J. Migraine is a food-allergic disease. Lancet. 1984 Sep 29;2(8405):719-21).

The spectrum of the light source?

Visible light: 380 to 740 nanometers (430–770 THz). Now indicated in the corresponding legend.

Page 4. (TTX) Na+ currents ◊ (TTX) sensitive Na+ currents

Thanks. Corrected.

An outstanding contribution that substantially advances our understanding of medicine induced headache.

Thank you very much for your support and helpful comments.

Reviewer #2 (Remarks to the Author):

The authors are investigating the role of Nav1.9 in medication over-use headache which is an important clinical problem. They develop a model of MOH based on chronic dosing with sumatriptan and suggest a mechanism whereby this results in reduced expression of PKAC-α, this is associated with enhanced coupling between NO and Nav1.9, trigeminal neuron hyper-excitability and behavioural hypersensitivity to mechanical stimuli, photo and phonophobia. The model is definitely interesting, the identification of Nav1.9 as a key hub in MOH would be a significant advance for the field. There are however a number of important weaknesses that need to be addressed:

We would like to thank Reviewer#2 for his/her very constructive comments. We have performed additional experiments to address all the points raised by Reviewer#2.

Major points:
1. Gender: All the mice used in the study were male yet as the authors point out migraine/MOH is much commoner in females. Why were female mice not studied? This is a significant deficiency in the study and at least the major finding that Nav1.9 has a key role in MOH (at a behavioral level) should be replicated female mice.

This is an important point, indeed, which has been raised by the 3 referees and the Editors. It is something we had in mind because the male: female ratio is about 1: 3.

We have now completed a new series of experiments in which the effects of chronic infusion of sumatriptan (or NaCl) and injection of SNP (or NaCl) at day 21 were tested on WT (n=20) and Nav1.9 KO (n=19) mouse females. As before, this series of experiments was made blind.

We treated chronically mouse females with sumatriptan (0.6 mg/kg/day, as with males) and assessed quantitatively generalized mechanical allodynia using von Frey filaments. We found that sumatriptan infusion but not saline solution (vehicle, 0.9%), decreased withdrawal thresholds to tactile stimuli applied to the hind paws of WT female mice. Females appeared more sensitive than males as hind paw withdrawal threshold was sensibly lower and sensory threshold was slower to recover. Similar to males, sumatriptan-treated WT females showed enhanced mechanical hypersensitivity at day 21 in response to SNP injection (0.03 mg/kg). This heightened allodynia was fully prevented in Nav1.9 KO female mice.

These data are now included in the result section on page 6 and in supplementary Figure 2B-D; it reads “*Because females have increased risk of developing migraine and MOH, we tested*

whether infusion of sumatriptan for 6 days (0.6 mg/kg/day) produced mechanical hypersensitivity and latent sensitization to NO in WT female mice as observed for the opposite gender. Chronic sumatriptan produced a strong reduction in mechanical withdrawal thresholds of the hind paw relative to saline-treated WT female mice and to sumatriptan-treated Nav1.9$^{-/-}$ female mice (Supplemental Fig. 2B). Injection of SNP (0.03 mg/kg) at day 21 once sensory thresholds had returned to pre-sumatriptan baseline values caused significantly stronger mechanical allodynia in sumatriptan-treated WT female mice compared to saline-treated WT female mice (Supplemental Fig. 2C). SNP-induced heightened mechanical allodynia was absent in sumatriptan-treated Nav1.9$^{-/-}$ female mice (Supplemental Fig. 2D), reaching similar amplitude to that caused by SNP in saline-treated Nav1.9$^{-/-}$ female mice (Supplemental Fig. 2D). This series of experiments shows that Nav1.9, as observed in male mice, had no role in SNP-induced allodynia in saline-treated animals, but contributes to the heightened SNP allodynia in sumatriptan-treated female mice."

2. Behavioral analysis and blinding: In the behavioral testing (eg. Fig 2). Why is a normalized mechanical withdrawal threshold given, what is it normalized to? The authors refer to a previous publication but this gives absolute values not normalized data. It is preferable in my view to give absolute values or at least a more detailed and transparent explanation (eg. is this because Nav1.9 -/- have different baseline values?). Peri-orbital testing data is only shown at day 21 given that this is the most relevant aspect for MOH/migraine it should also be given at earlier timepoints (as it has been for hindpaw).

We apologize for the ambiguity. We refer to the paper mainly for the use of von Frey filaments. The first reason of using normalized threshold is because of within-strain variation of mechanical withdrawal threshold even in standardized mouse environment. For example, WT male mice have mechanical withdrawal threshold ranging from 0.4 to 1.5 g. Plotting absolute values makes the graphs messy and hard to figure out. Data points were normalized to Day 0 (the measure obtained just before the implantation of the minipump, e.g. Figure 2A,C) or H0 (the measure made just before the injection of SNP, e.g. Figure 2B,D). This is now indicated in the methods Section on page 15. This also helps comparison between genotypes. However, it is important to note that Nav1.9 KO mice have mechanical withdrawal threshold (evaluated using von Frey filaments) not different from WT mice, as Nav1.9 has no apparent role in mechanical withdrawal threshold under normal conditions. This has been published previously by different groups (Amaya et al. 2006. J. Neurosci. 26:12852–12860 ; Priest et al. 2005. Proc. Natl. Acad. Sci. USA. 102:9382–9387; Lolignier et al., Cell Rep. 2015 May 19;11(7):1067-78; etc..). However, recently Hoffmann et al., (2017, Pain. 158:58-67) suggested that deletion of Nav1.9 reduces noxious sensory thresholds.

About peri-orbital testing: these experiments are really difficult in mice. We have failed to measure peri-orbital thresholds every day (or every 2 days) because mice tend to exhibit increased nocifensive behavior when stimulated repetitively days after days with von Frey filaments in the peri-orbital region. They become very agitated as soon as we move forward the filaments, so it was technically not possible to apply the different filaments onto the face.

Re. blinding in the reporting section the statement is that 'The behavioral experiments in which animals were treated with sumatriptan or saline solution chronically (with minipumps), and injected at day 21 with SNP or vehicle were made double blind; i.e. the investigator was not aware of the content of the minipump, nor was he aware of the solution (SNP or vehicle) he was injecting on day 21.' I was confused by this as All behavioral experiments should be performed blind. Was the investigator also blind to genotype? A statement regarding blinding needs to be made in the manuscript (please note that it doesn't make sense referring to a double blind experiment in animals.

Our mistake. The investigator was not aware of the content of the minipump, neither of the solution he/she was injecting (SNP or saline solution) on day 21. However, the investigator was aware of the genotype. The text in the reporting section was corrected and a sentence has been added in the method section on page 15.

3. Mechanism by which chronic sumatriptan enhances coupling between NO and Nav1.9: Is there direct evidence that chronic sumatriptan treatment enhances NO release and there are also assays available for assessing cGMP? There is a suggestion that the sumatriptan leads to reduced PKAC-α and this results in less inhibition of cGMP signalling. It would be helpful to look at this further at protein level for instance with western blot analysis to back up the q-PCR data and also looking at the activated phospho T197 PKA form which is currently only examined with immunostaining.

Chronic sumatriptan treatment has been shown to increase the level of expression of the NO synthase in trigeminal ganglion dural afferents (De Felice et al., 2010, Brain. 133, 2475-2488; cited in the ms), so there is potential for increased NO release, although this has not been directly tested.

Our data show sumatriptan-treated mice have lower levels of PKA transcripts and reduced PKA subunit phospho T197 immunostaining in meningeal TG neurons compared to saline-treated mice (page 9). Further to Reviewer #2 suggestion, we made a new series of experiments in order to evaluate phospho T197 PKA expression in TGs using western blots. Changes in PKA expression in TGs from sumatriptan-treated mice (8 mice, 16 TGs) were evaluated by quantifying band intensities on phospho T197 PKA blots and comparing to blot band intensities in saline-treated mice (8 mice, 16 TGs) as controls. Densitometry analysis of background-subtracted blots from 20 μg of total lysate showed a 22% decrease in phospho T197 PKA expression in TGs from sumatriptan-treated mice versus controls (see new Supplemental Fig. 8C). However, this decrease did not reach significant level (Mann-Whitney test) due to sample variability (see Supplemental Fig. 8C,D). These data are now presented on page 9 and supplemental Figure 8.

We also normalized intensities of phospho T197 PKA blots to GAPDH blots (data not shown). Normalization of mean PKA signals to GAPDH ones shows a 15 ± 2% decrease in T197 PKA expression in SUMA-treated mice (4 mice, 8 TGs) compared to saline-treated mice (4 mice, 8 TGs). This again did not reach statistically significant level (Mann-Whitney test).

Overall, we think it may be difficult to detect a significant decrease in PKA protein level in whole TGs using Western blot, especially if the downregulation affects a subset of TG neurons.

4. The level of statistical reporting could be improved throughout: See comments on blinding above. In the methods section some general comments on the statistical tests used are provided. However it is essential that the exact test used for that specific experiment is given in each case (ie in the actual results we don't know when a t-test, versus one way versus two way ANOVA was used etc). In 'A description of any assumptions or corrections, such as tests of normality and adjustment for multiple comparisons'. NA is given. The data should always be tested for normality and I presume that this was done? This should be stated in the statistics section.

We now mention in the figure legend the statistical test used for each analysis. We clarified the statistics in the method section and in the Reporting Summary. It reads "*All values are shown as mean ± standard error of the mean (SEM) and n represents the number of animals or cells examined. Except for some behavioral experiments, no statistical methods were used to pre-determine sample sizes but our sample sizes are similar to those reported in previous publications. Assessment of normality for sample >14 was tested using the Kolmogorov-Smirnov or the D'Agostino-Pearson Omnibus K2 normality test. Tests for differences between two normally distributed populations were performed using two-tailed t-test. Small sample size lacks power to test normality, therefore we typically used non-parametric Mann–Whitney test and Wilcoxon's test to test for differences between two populations in small samples ($n \leq 15$). Two-way (repeated measures) ANOVA followed by Student-Newman-Keuls multiple comparison procedure was used for experiments with multiple groups and two dependent variables. Figure legends specify which test was used for specific experiments. Significant levels were set at $p \leq 0.05$. Analysis used a combination of Clampfit 9.2 (Molecular Devices), Origin 7.0 (OriginLab) and PRISM 7.0 (GraphPad) softwares*."

The sample size chosen relates to the variance in the data, the effect size that we think is meaningful as well as the significance level (alpha). According to the ARRIVE guidelines There should be a power calculation performed at the outset to justify sample size.

We did not systematically performed calculation to justify sample size. However, in some behavioral experiments we performed sample size calculations using the software G*Power (http://www.gpower.hhu.de/). We found that sample sizes with a minimum of 8-13 animals were necessary in most experiments, hence our typical sample sizes of 10-15 animals in most tests.

Minor points

1. Supplemental figure 3 it looks to me that the hypersensitivity induced by sumatriptan is less in the Nav1.8-/- versus WT mice (panel A). What is the result of direct statistical comparison of these two groups?

There is indeed a significant difference during the 'recovery' phase at day 10 and day 12 between Nav1.8 KO mice versus WT mice treated with sumatriptan (Supplemental Figure 3A; *$p<0.05$, compared to Nav1.8 KO SUMA with Mann-Whitney non-parametric test). We know that sumatriptan has no acute effect on TG Nav1.8 current and that Nav1.8 transcript level remains stable upon chronic sumatriptan treatment. Therefore, it is plausible that

Nav1.8 contributes to maintain hyperexcitability of TG neurons, given its role (along with Nav1.7) in the generation/propagation of spikes in nociceptors.

2. I may have missed it but more information needs to be given re mice expressing β-Gal at the SCN11a locus. At least a reference of where/how this line was generated should be given (this applies to all the transgenic lines).

The Scn11a-GAL reporter mouse was made by JSK and published in 2007 (ref 19). An IRES/LacZpA cassette followed by a loxP/neo/loxP cassette was inserted into the end of exon 5 of the Scn11a gene. Reference to previously published papers is now indicated in the method section. The Nav1.9 and Nav1.8 KO transgenic lines are gifts from Prof N. J. Wood and were previously published (ref 19, 23). We added the references in the method section.

The authors state that double staining of CGRP and Nav1.9 could not be undertaken. Good antibodies to β Galactosidase are available meaning that it would be simple to assess both the co-expression of Nav1.9 immunostaining with β Gal and also β Gal and CGRP.

This is a good suggestion. Unfortunately the two anti-β-Galactosidase polyclonal antibodies we tested on TGs showed strong background level. So, we decided instead to perform immunostaining of CGRP in TGs from *Scn11a*-GAL reporter mice, and then to reveal β-gal enzymatic activity. This required to remove glutaraldehyde from the fixative solution and once revealed to post-fix the tissue for 2h with 2% PFA. This approach is now described in the method section on page 20. We found that the quasi-totality of CGRP-positive TG neurons exhibited β-gal staining in cryosections from Scn11a-GAL reporter transgenic mice (n = 63/64). These data are now presented on page 5 and Supplemental Figure 1B; they indicate/confirm that CGRP is expressed in Nav1.9-positive TG neurons.

3. In results page 7. 'Neither the mean Nav1.9 peak current amplitude (Fig 4B), nor the level of Nav1.9 mRNA…' Do they mean to say 'Neither the mean Nav1.9 peak current density?' which is the top graph in the panel?

Yes, we meant 'current density'. This has been corrected on page 8 of the revised ms.

4. In current clamp analysis of dural afferents in Figure 5. Do any of these neurons develop spontaneous activity in response to SNP? Is there a change in action potential waveform in response to SNP?

We did not see changes in AP waveform. Spontaneous firing in response to SNP application was also a very rare event. SNP occasionally caused small depolarization, but did not generate firing unless the membrane potential was near the negative slope conductance (regenerative process) of the Nav1.9 current. SNP-induced small depolarization may be linked to TRPA1/V1 activation.

Reviewer #3 (Remarks to the Author):

Bonnet et al. report the role of the voltage-gated sodium channel Nav1.9 in medication overuse headache (MOH). Nav1.9 is a channel involved in pain sensation and generates a persistent sodium current to modulate neuronal activity. It´s mutations are associated with different human pain disorders. In mice, the authors show here that chronic use of triptans induces MOH through abnormal activation of meningeal Nav1.9 channels. Bonnet and colleagues administer the 5-HT receptor agonist sumatriptan, which is approved for acute therapy in migraine and in cluster headache, and show that animals chronically treated with the drug display increased responsiveness / abnormal activation of Nav1.9 to nitric oxide (NO). NO is a well-known trigger for headache and migraine. Phenotypic readout for the "migraine-like symptoms" in mice are generalized allodynia, photophobia, and phonophobia. The authors suggest that Nav1.9-mediated release of neuropeptides from meningeal nociceptors involves degranulation of mast cells and causes inflammatory pain.

This is a very interesting paper dealing with a highly relevant topic and has implications for translational approaches in human pain therapy. The findings are new. Methods and experimental approaches are state-of-the-art and I have only few minor suggestions:

We would like to thank Reviewer #3 for his/her very helpful and positive comments.

Migraine occurs at a 3:1 female to male ratio. In their experiments, the authors report the results from male mice. Can the authors maybe comment whether sex-specific differences have also been observed using female mice? This would be a very interesting point to address, however, repeating all experiments with a cohort of female mice would exceed the aim of the present study.

This is an important point, indeed, which has been raised by the 3 referees and the Editors. It is something we had in mind because the male: female ratio is about 1: 3.

We have now completed a new series of experiments in which the effects of chronic infusion of sumatriptan (or NaCl) and injection of SNP (or NaCl) at day 21 were tested on WT (n=20) and Nav1.9 KO (n=19) mouse females. As before, this series of experiments was made blind.

We treated chronically mouse females with sumatriptan (0.6 mg/kg/day, as with males) and assessed quantitatively generalized mechanical allodynia using von Frey filaments. We found that sumatriptan infusion but not saline solution (vehicle, 0.9%), decreased withdrawal thresholds to tactile stimuli applied to the hind paws of WT female mice. Females appeared more sensitive than males as hind paw withdrawal threshold was sensibly lower and sensory threshold was slower to recover. Similar to males, sumatriptan-treated WT females showed enhanced mechanical hypersensitivity at day 21 in response to SNP injection (0.03 mg/kg). This heightened allodynia was fully prevented in Nav1.9 KO female mice.

These data are now included in the result section on page 6 and in supplementary Figure 2B-D; it reads "*Because females have increased risk of developing migraine and MOH, we tested whether infusion of sumatriptan for 6 days (0.6 mg/kg/day) produced mechanical hypersensitivity and latent sensitization to NO in WT female mice as observed for the*

opposite gender. Chronic sumatriptan produced a strong reduction in mechanical withdrawal thresholds of the hind paw relative to saline-treated WT female mice and to sumatriptan-treated Nav1.9$^{-/-}$ female mice (Supplemental Fig. 2B). Injection of SNP (0.03 mg/kg) at day 21 once sensory thresholds had returned to pre-sumatriptan baseline values caused significantly stronger mechanical allodynia in sumatriptan-treated WT female mice compared to saline-treated WT female mice (Supplemental Fig. 2C). SNP-induced heightened mechanical allodynia was absent in sumatriptan-treated Nav1.9$^{-/-}$ female mice (Supplemental Fig. 2D), reaching similar amplitude to that caused by SNP in saline-treated Nav1.9$^{-/-}$ female mice (Supplemental Fig. 2D). This series of experiments shows that Nav1.9, as observed in male mice, had no role in SNP-induced allodynia in saline-treated animals, but contributes to the heightened SNP allodynia in sumatriptan-treated female mice."

Can the authors speculate whether the application of currently available sodium-channel blockers in combination with sumatriptan or other headache medications would be beneficial? Yes?

It is an important point indeed and a strategy we are currently developing. As usual, the difficulty resides in the specificity of the inhibitor. We amended the text on page 14 to read: "*Therefore, the use of Nav1.9 channel inhibitors, in combination with sumatriptan or other headache medications, may represent a new acute and preventive option for migraine treatment*".

Methods: Please provide more details on the generation of the Nav1.8 and Nav1.9 KO-mice used in the study.

We now provided this information. Reference to previously published papers using these mice is now indicated in the method section. The Scn11a-GAL reporter mouse was made by JSK and published in 2007 (ref 19). An IRES/LacZpA cassette followed by a loxP/neo/loxP cassette was inserted into the end of exon 5 of the Scn11a gene. The Nav1.9 and Nav1.8 KO transgenic lines are gift from Prof N. J. Wood and were previously published (ref 19, 23).

REVIEWERS' COMMENTS:

Reviewer #1 (Remarks to the Author):

The authors have provided new data and well reasoned discussion of the points raised in the intial review. I have but just one suggestion. While the authors nicely executed parallel experiments in female mice, as suggested by all 3 reviewers, they do not npte in the added text that the effect in females was greater than in males, which nicely parallels the sexual dimorphism found in migraine in humans.

Reviewer #2 (Remarks to the Author):

The authors have done an excellent job of revising the manuscript and addressing my concerns I particularly commend the addition of a female cohort of mice.

Reviewer #3 (Remarks to the Author):

All points have been sufficiently addressed.

NCOMMS-18-36489A Point-by-point reply to Reviewers' comments.

We thank the referees for his/her very helpful comments.

Reviewer #1 (Remarks to the Author):

The authors have provided new data and well reasoned discussion of the points raised in the initial review. I have but just one suggestion. While the authors nicely executed parallel experiments in female mice, as suggested by all 3 reviewers, they do not note in the added text that the effect in females was greater than in males, which nicely parallels the sexual dimorphism found in migraine in humans.

We added a comment on page 13 (discussion), it reads: "*Importantly, we found that hypersensitivity to SNP was greater in MOH female than in male mice, which parallels the sexual dimorphism* reported *in MOH and migraine in humans*."

Reviewer #2 (Remarks to the Author):

The authors have done an excellent job of revising the manuscript and addressing my concerns I particularly commend the addition of a female cohort of mice.

Reviewer #3 (Remarks to the Author):

All points have been sufficiently addressed.
